# Extracellular vesicles as indicators of environmental stress response in *Lactiplantibacillus plantarum*

**DOI:** 10.20517/evcna.2026.26

**Published:** 2026-05-29

**Authors:** Agnieszka Razim, Astrid Laimer-Digruber, Anna M. Schmid, Tanja V. Edelbacher, Magdalena E. Paschall, Tamara Weinmayer, Michael Thaler, Rim Kebreab, Magdalena E. Skalska, Paweł Migdał, Mattia Morandi, Catherine Daniel, Dagmar Srutkova, Martin Schwarzer, Jiri Hrdy, Monika Ehling-Schulz, Sabina Górska, Aleksandra Inic-Kanada, Ursula Wiedermann, Irma Schabussova

**Affiliations:** ^1^Institute of Specific Prophylaxis and Tropical Medicine, Center for Pathophysiology, Infectiology and Immunology, Medical University of Vienna, Vienna 1090, Austria.; ^2^Hirszfeld Institute of Immunology and Experimental Therapy, Polish Academy of Sciences, Wrocław 53-114, Poland.; ^3^Institute of Microbiology, Centre for Pathobiology, Department of Biological Sciences and Pathobiology, University of Veterinary Medicine, Vienna 1210, Austria.; ^4^Medical Physics Department, Marian Smoluchowski Institute of Physics, Jagiellonian University, Kraków 30-348, Poland.; ^5^Institute of Organic Chemistry and Biochemistry, Czech Academy of Sciences, Prague 160 00, Czech Republic.; ^6^Univ. Lille, CNRS, Inserm, CHU Lille, Institut Pasteur Lille, U1019 - UMR 9017 - CIIL - Center for Infection and Immunity of Lille, Lille F-59000, France.; ^7^Laboratory of Gnotobiology, Institute of Microbiology, Czech Academy of Sciences, Nový Hrádek 549 22, Czech Republic.; ^8^Institute of Clinical Immunology and Allergology, First Faculty of Medicine, Charles University and General University Hospital in Prague, Prague 121 11, Czech Republic.

**Keywords:** EVs, probiotics, stress, gut environment, bacterial communication, Fourier-transform infrared spectroscopy, ultracentrifugation

## Abstract

**Aim:** To determine how exposure to bile influences the production, physicochemical properties, and molecular composition of extracellular vesicles (EVs) released by the probiotic *Lactiplantibacillus plantarum* (*L. plantarum*) NCIMB 8826.

**Methods:** *L. plantarum* cultures were incubated with and without a physiologically relevant cocnentartion of bile. EVs were isolated by ultracentrifugation followed by size-exclusion concentration (SEC) and characterized per Minimal Information for Studies of Extracellular Vesicles guidelines. Assessments included purity [protein and peptidoglycan (PGN) content], particle size distribution, zeta potential (ZP), lipid profiling, stability assays under varying pH, ionic strength (NaCl) and detergent challenge, Fourier-transform infrared spectroscopy (FTIR), and proteomic analysis were performed. Comparisons were made between purified EVs and parent bacterial cells, and between EVs from bile-exposed and control conditions.

**Results:** SEC markedly reduced copurifying proteins and PGNs, improving EV compositional purity. Purified *L. plantarum* EVs (LpEVs) displayed surface lipid profiles and ZP that were distinct from parent cells and maintained stability across a range of pH values, elevated NaCl, and low detergent concentrations. Bile exposure induced release of larger LpEVs enriched in proteins involved in bile metabolism. FTIR detected bile-induced molecular changes in LpEVs that differed from alterations observed in whole bacterial cells.

**Conclusion:** *L. plantarum* modulates EV size and cargo in response to bile stress, producing stable, compositionally distinct vesicles enriched in bile salt hydrolase. These findings indicate that bacterial EVs act as dynamic responders to environmental stress and may mediate early host–microbe communication prior to detectable cellular changes.

## INTRODUCTION

Bacterial extracellular vesicles (EVs) are lipid bilayer–enclosed particles secreted by bacteria, typically spherical in shape and ranging from 20 to 400 nm in diameter^[1]^. They carry specific cargo such as proteins, lipids, and nucleic acids, with their composition varying depending on whether they originate from Gram-positive or Gram-negative bacteria^[2]^. Microbe-associated molecular patterns, recognized by pattern recognition receptors, are present in both the parent bacteria and their EVs. These include lipopolysaccharide [LPS; ligand for Toll-like receptor 4 (TLR4)], lipoproteins (TLR2 ligands), peptidoglycans (PGNs; ligands for nucleotide-binding oligomerization domain–containing proteins NOD1 and NOD2), and nucleic acids such as DNA and small RNAs (sRNAs; TLR7 and TLR9 ligands)^[3]^.

Because of their small size, EVs can penetrate host tissues and disseminate throughout the body more readily than whole bacteria^[4]^, enabling both local and systemic modulation of host physiology. Notably, this size advantage can confer functional superiority over the parent organism: for instance, *Bacteroides fragilis* EVs drive IL-10–producing regulatory T cells more efficiently than whole bacteria^[5]^, and EVs from the gut commensal *Akkermansia muciniphila* have been shown to cross the intestinal barrier and reach systemic circulation, exerting metabolic and immunomodulatory effects at distant sites^[6]^. At the epithelial interface, bacterial EVs act as potent modulators of barrier function and inflammatory signaling. *Escherichia coli* (*E. coli*) C25–derived EVs induce a dose-dependent inflammatory response in intestinal epithelial cells (IECs)^[3]^, while *Fusobacterium nucleatum* EVs trigger apoptosis in IECs and Caco-2 cells, leading to disruption of epithelial barrier integrity and increased oxidative stress^[7]^. Bacterial EVs also directly regulate immune cells: EVs from the probiotic *E. coli* O83 exert immunomodulatory effects on mouse and human airway immune cells^[8]^, and *Bacteroides fragilis* EVs selectively promote regulatory T cell differentiation^[5]^. Beyond these local effects, EVs are capable of long-distance immune regulation. *Vibrio cholerae* EVs, for example, alter microRNA expression in eukaryotic cells at distal sites, thereby suppressing host immune responses^[9]^. Together, these findings establish bacterial EVs as versatile and potent intercellular messengers that can operate across multiple biological scales, from mucosal surfaces to systemic compartments.

The diverse biological activities and molecular composition of bacterial EVs suggest that they fulfill multiple roles in microbial ecology and host interactions, encompassing colonization, nutrient acquisition, antimicrobial defense, and interspecies communication^[10]^. A key but incompletely understood aspect of EV biology is the extent to which their production and composition are shaped by environmental conditions. Evidence from multiple systems indicates that EV release is not constitutive but dynamically regulated: in marine bacteria, for instance, both the size and rate of EV production vary within the same strain in response to nutrient availability, temperature, and light exposure^[11]^. Similarly, environmental nutrient status influences EV function in the gut. Under nutrient-deficient conditions, *Bacteroides thetaiotaomicron* produces EVs equipped with high-affinity vitamin B12-binding proteins that scavenge this micronutrient from the environment, and these vesicles are selectively internalized by the parent bacteria or host cells^[12]^.

Despite substantial progress in the study of bacterial EVs, significant knowledge gaps remain. The precise mechanisms by which environmental stressors trigger EV biogenesis and release are not yet fully understood. Changes in size and cargo determine how EVs are sensed, taken up, and responded to by host cells, so stress-induced changes in EVs production are expected to alter host exposure to microbial structures and metabolites, thereby reshaping immune and metabolic interactions^[13]^. The evolutionary significance of EV production under stress also remains unclear, particularly regarding how it may confer adaptive advantages in different ecological niches. Therefore, there is an urgent need to develop and refine advanced methodologies for the isolation, characterization, and functional analysis of bacterial EVs not only from artificial culture systems but also under conditions that more closely mimic their natural environments. Clarifying these relationships is essential for interpreting bacterial EV functions *in situ* and for harnessing them therapeutically.

In this study, we used *Lactiplantibacillus plantarum* (*L. plantarum*) NCIMB 8826, a strain originally isolated from human saliva, known for its exceptional adaptive capabilities and parental to the well-studied *L. plantarum* WCFS1^[14]^, as a model to investigate the impact of stress on EV production. We isolated *L. plantarum* EVs (LpEVs) and characterized them according to the Minimal Information for Studies of Extracellular Vesicles (MISEV) guidelines^[15]^. Size-exclusion concentration (SEC) was performed for purification, which markedly reduced the contents of contaminating proteins and PGNs. Compared with the parent bacteria, LpEVs exhibited distinct surface lipid profiles and zeta potential (ZP). Stability assays demonstrated that LpEVs were highly resistant to variations in pH, elevated NaCl concentrations, and sodium dodecyl sulfate (SDS) exposure. Notably, LpEVs produced in medium supplemented with 0.25% bile showed increased particle size and protein content, including enrichment in bile metabolism–associated proteins such as bile salt hydrolase (BSH). Fourier-transform infrared spectroscopy (FTIR) further revealed bile-induced spectral changes in LpEVs, reflecting alterations in protein and fatty acid composition under stress. Proteomic analysis and FTIR spectroscopy collectively revealed that exposure to bile induced more pronounced compositional changes in LpEVs than in the parent bacteria. These findings suggest that LpEVs function as indicators of environmental stress, reflecting adaptive responses that occur even before such changes become detectable at the cellular level.

## METHODS

### EV production and purification

**Figure 1:**
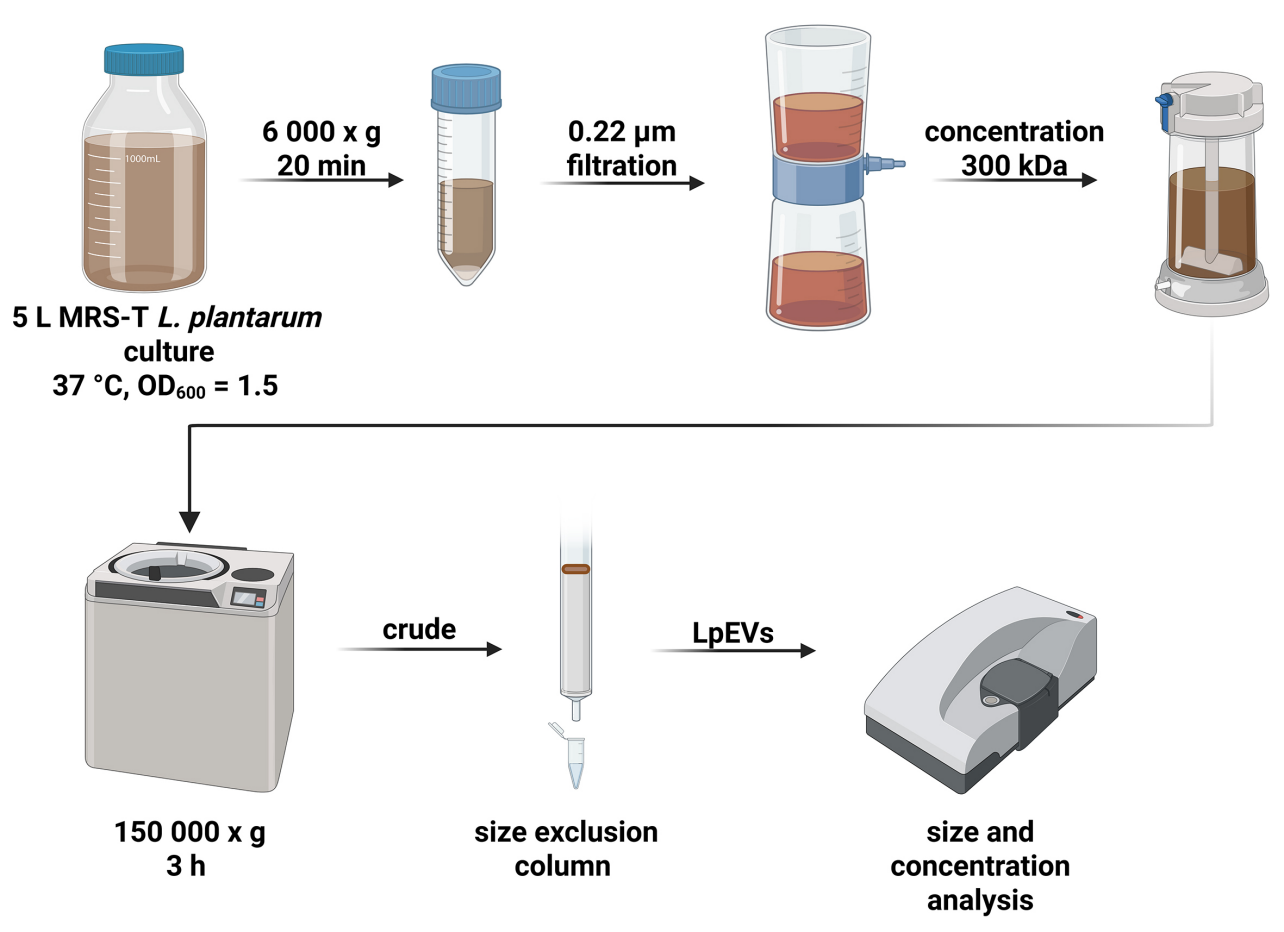
Production and purification of LpEVs. *L. plantarum* was grown in MRS-T medium at 37 °C in a closed bottle until an OD600 of 1.5 was reached. The culture was centrifuged, filtered, concentrated, and subjected to UC. Crude LpEVs were further purified via SEC, pooled, and characterized. Figure created with BioRender (https://biorender.com/l3z5dk4). LpEVs: *L. plantarum* EVs; *L. plantarum*: *Lactiplantibacillus plantarum*; MRS-T: De Man–Rogosa–Sharpe broth supplemented with Tween 80; UC: ultracentrifugation; SEC: size-exclusion concentration.

EVs were isolated [Figure 1] from *L. plantarum*^[16]^, kindly provided by Dr. Daniel (Institut Pasteur de Lille, France). Cultures were grown in 5 L of De Man–Rogosa–Sharpe (MRS; Merck, Germany) broth supplemented with Tween 80 (MRS-T) in closed bottles without shaking at 37 °C (Innova 4230 Incubator, Eppendorf New Brunswick, Germany). The culture was inoculated with an overnight starter culture at a 1:200 ratio and incubated until an optical density at 600 nm (OD600) of 1.5 was reached. OD600 = 1.5 corresponds to the exponential growth phase of *L. plantarum* [Supplementary Figure 1] and was selected to obtain robust vesicle yield while minimizing contamination from membrane fragments released during stationary-phase cell lysis. Bacterial cells were then removed via centrifugation (6,000 × *g*, 20 min, 4 °C; Heraeus, Thermo Fisher Scientific, USA), followed by filtration through a 0.22-µm sterile vacuum bottle-top filter (Steritop, Merck). The resulting cell-free supernatant was concentrated approximately 13-fold using an Amicon Ultra Stirring Cell (Merck) equipped with 300 kDa ultrafiltration discs.

Crude LpEVs were obtained via ultracentrifugation (UC) of the concentrated supernatant at 150,000 × *g* for 3 h at 4 °C (Beckman Coulter, USA; 45Ti rotor) and resuspended in 500 µL of 25 mM 4-(2-hydroxyethyl)-1-piperazineethanesulfonic acid buffer containing 0.15 M NaCl (HEPES-N). The EVs were further purified via SEC using qEVoriginal/35 nm Gen 2 columns coupled to an Automatic Fraction Collector (IZON Science, France), following the manufacturer’s instructions. Eight fractions of 400 µL each were collected [Supplementary Figure 2A and B]. Fractions enriched in homogeneous particles - typically corresponding to the purified collection volume (PCV) 1.1-1.3 - were pooled and stored at 4 °C for immediate use or at -20 °C for long-term storage.

Mock EVs were prepared following the same procedure, using uninoculated 5 L of MRS-T medium. After incubation at 37 °C for 8 h (the time required for bacterial cultures to reach an OD600 of 1.5), the medium was processed in parallel with the inoculated samples, including centrifugation, filtration, concentration, UC, SEC, and fraction pooling (PCV 1.1-1.3).

### Characterization of EVs

#### Size, concentration, and ZP measurements

Particle size and concentration were measured using Zetasizer Ultra Red Label (Malvern Panalytical, UK) as previously described^[8]^. The working ranges were 1 × 10^8^ - 1 × 10^12^ particles/mL for concentration and 0.3 nm - 15 µm for diameter. Analyses were performed in a quartz batch cuvette (ZEN2112, Malvern Panalytical) using General Mode, with all measurements conducted in triplicate (technical replicates). ZP was determined using a disposable folded capillary cell (DTS1070, Malvern Panalytical)^[8,17]^. EV samples were diluted 1:500 in 1 mM HEPES, whereas bacterial samples from overnight cultures were diluted 1:1,000 in 1 mM HEPES. Data quality and analysis were assessed using Malvern ZS Xplorer software (version 3.2.0.84). Cryogenic electron microscopy (cryo-EM) was additionally employed for EV size validation.

#### Transmission electron microscopy

LpEVs were negatively stained as previously described^[8]^. Briefly, 5 µL of the EV suspension was adsorbed onto formvar/carbon-coated grids for 20 s, stained with 5-10 µL of 1% aqueous uranyl acetate, and blotted twice using the single-drop method. The grids were air-dried and imaged using an FEI Tecnai 20 transmission electron microscope (FEI, Netherlands) equipped with a 4K Eagle CCD camera. Images were processed using Adobe Photoshop.

#### Cryo-EM

For cryo-EM analysis, LpEVs were applied to Quantifoil EM grids (Electron Microscopy Sciences, USA) pre-coated with 10-nm protein A–conjugated colloidal gold particles (Au–NP; Aurion, Netherlands), following an established protocol^[8]^.

### Analysis of surface composition

#### Surface lipids

Surface lipid analysis was performed using time-of-flight secondary ion mass spectrometry (ToF-SIMS) as previously described^[8]^. Briefly, surface lipids of LpEVs and *L. plantarum* were analyzed with a ToF-SIMS V instrument (ION-TOF GmbH, Münster, Germany) equipped with a 30 keV Bi^3+^ ion beam (~0.61 pA). Positive secondary ions (m/z 1-900) were collected from 150 µm × 150 µm areas, and spectra were internally calibrated using standard hydrocarbon peaks. The Bi^3+^ beam was rastered over a 128 × 128 pixel region, and ion doses were maintained below the static limit (1 × 10^12^ ions/cm^2^) for all analyses. Surface charge neutralization was achieved using a low-energy electron flood gun. The data supporting this analysis are available at ROD BUK, Jagiellonian University in Kraków: https://uj.rodbuk.pl/dataset.xhtml?persistentId=doi:10.57903/UJ/GLBXEG.

#### Lipoteichoic acid

*L. plantarum* (10 µg of cell lysate), LpEVs (1.5 × 10^10^ particles), and commercial lipoteichoic acid (LTA, 10 µg; Sigma-Aldrich, USA) were mixed with 4× Laemmli buffer, boiled for 5 min at 96 °C, and separated on a 4%-12% gradient SDS–PAGE gel (Thermo Fisher Scientific, USA). Proteins were transferred onto a polyvinylidene fluoride membrane (Invitrogen, USA) at 30 V for 1 h, blocked with 3% bovine serum albumin (BSA) for 1 h at room temperature (RT), and probed with an anti-LTA antibody (ABAAb02033-1.1, Szabo-Scandic, Austria; 1:500, 2 h, RT). After washing, the membrane was incubated with an alkaline phosphatase–conjugated anti-mouse IgG1 antibody (Sigma-Aldrich, USA; 1 h, RT). The signal was developed using NBT/BCIP (Sigma-Aldrich, USA) and stopped by washing with water. The membrane was imaged using a Gel Doc system (Bio-Rad, USA).

#### PGN

PGN quantification was performed as described by Bitto *et al.*^[18]^. Crude and purified LpEV samples (2.5 × 10^10^ particles in 25 µL) were treated with 100 µL of 1 M NaOH for 30 min at 38 °C, followed by acid hydrolysis with 125 µL of 0.5 M H_2_SO_4_ and 1,250 µL of concentrated H_2_SO_4_. Samples were then boiled for 5 min and rapidly cooled under running water. Subsequently, 12.5 µL of CuSO_4_ and 25 µL of 1.5% 4-phenylphenol (Sigma-Aldrich, USA) in 96% ethanol were added and incubated for 30 min at 30 °C. Absorbance was measured at 560 nm in 96-well plates using a plate reader (Tecan Trading AG, Switzerland). Each test was done in duplicate (technical repeats) for each of the three tested batches (three biological repeats).

### Analysis of EV storage stability

The long-term storage stability of LpEVs was evaluated following a modified protocol of Görgens *et al.*^[19]^. After UC (150,000 × *g*, 3 h; SW41Ti rotor), LpEVs were resuspended in one of five buffers: (i) 25 mM HEPES with 0.9% NaCl (HEPES-N); (ii) 25 mM HEPES with 0.9% NaCl and 0.2% BSA (HEPES-NA); (iii) phosphate-buffered saline (PBS); (iv) PBS with 0.2% BSA (PBS-A); or (v) PBS with 25 mM HEPES and 0.2% BSA (PBS-HA). Aliquots were prepared in triplicate and stored at 4, -20, or -80 °C. Samples were analyzed at baseline (time 0) and after 1, 6, and 12 months. Each aliquot was thawed only once for size and concentration measurements using the Zetasizer; after measurement it was discarded. All measurements were performed from a single stock preparation to ensure consistency across analyses.

### Analysis of environmental stability of bacteria and their EVs

The physical stability of *L. plantarum* and LpEVs was evaluated under varying salt, pH, and detergent conditions relevant to the human physiological environment. Experiments were performed in triplicate (biological replicates).

**Salt stability**: LpEVs (1.2 × 10^11^ particles/mL in 50 µL of HEPES-N) were used as the starting material. Concentrated NaCl was added to achieve final concentrations of 0.25, 0.35, 0.5, and 1.0 M. Samples were mixed and analyzed for particle size and concentration within 30 min. In parallel, overnight bacterial cultures were diluted 1:200 in MRS-T supplemented with the same NaCl concentrations.

**pH stability**: LpEVs (0.5 × 10^10^ particles) were pelleted via UC (150,000 × *g*, 3 h; SW41 rotor), resuspended in 50 µL of HEPES-N adjusted to pH values of 5.7-8.2 (in 0.5-unit increments), and analyzed within 30 min as described above. In parallel, overnight bacterial cultures were diluted 1:200 in MRS-T adjusted to the same pH using citrate–phosphate buffer.

**Detergent stability**: LpEVs (1 × 10^11^ particles/mL in 90 µL of HEPES-N) were incubated with SDS at final concentrations of 0, 0.1, 0.6, 2.2, and 8.9 mM and analyzed within 30 min. In parallel, overnight bacterial cultures were diluted 1:200 in MRS-T containing the same SDS concentrations.

All experiments were repeated three times, and each sample was prepared in triplicate (technical replicates). Bacterial growth under all conditions was monitored by measuring OD600 at 0, 3, 5, 6, 7, and 8 h in 96-well plates using a plate reader. The OD600 value at time 0 was used as the baseline and subtracted from subsequent measurements.

### Bile stress assays

#### Bacterial growth and EV production

To evaluate the effect of bile stress, overnight *L. plantarum* cultures were diluted 1:200 in MRS-T supplemented with 0%-2% (twofold serial dilutions) bovine bile (Sigma-Aldrich, USA). For EV isolation, *L. plantarum* cultures were grown in 0.25% bovine bile (LpEVs^BILE^), following the same procedure as for standard LpEV preparation. Corresponding MOCK EVs (MOCK EVs^BILE^) were prepared from uninoculated medium processed in parallel. Assays were performed in triplicate (biological repeats).

#### Nucleic acid isolation and analysis

RNA was isolated using an RNA extraction kit (IZON Science, France), and RNase treatment was performed following a previously established protocol^[8]^. Briefly, 375 µL of LpEVs and LpEVs^BILE^ (~1.5 × 10^11^ EVs) were treated with proteinase K (100 µg/mL, 2 h, 37 °C; Thermo Fisher Scientific, USA), followed by enzyme inactivation at 75 °C for 1 h. Subsequently, RNase One (10 U/mL; Promega, USA) was applied for 30 min at 37 °C and inactivated with RNase inhibitor (SUPRAase·InTM, 1 U/mL; Thermo Fisher Scientific, USA). RNA was extracted in a final volume of 50 µL and analyzed using a Bioanalyzer (Agilent Technologies, USA).

DNA was isolated from 375 µL of the IZON fraction of LpEVs and LpEVs^BILE^ (~1.5 × 10^11^ EVs) using the FavorPrep Tissue Genomic DNA Extraction Kit (Favorgen Biotech Corporation, Taiwan). DNA concentration was measured using a NanoDrop 1000 spectrophotometer (Thermo Fisher Scientific, USA), which has a working range for dsDNA is 2-3,700 ng/µL, with a suggested minimum concentrations > 20 ng/µL. Samples were visualized on a 2% agarose gel stained with Midori Green (Thermo Fisher Scientific, USA).

#### Protein quantification and SDS–PAGE

Protein concentrations in LpEVs, LpEVs^BILE^, and the corresponding MOCK controls were determined using three independent assays: (i) Bradford assay (Bio-Rad, USA); (ii) bicinchoninic acid (BCA) assay (Thermo Fisher Scientific, USA); and (iii) Pierce 660 nm Protein Assay (Thermo Fisher Scientific, USA), following the manufacturers’ protocols. Sample volumes were 5 µL (Bradford), 25 µL (BCA), and 10 µL (Pierce). Absorbance was measured using a plate reader. Protein content was calculated as µg of protein per 1 × 10^11^ EVs. BSA standards were prepared in 25 mM HEPES-N. Data obtained from each method were analyzed using one-way analysis of variance (ANOVA), comparing LpEVs^BILE^ with LpEVs. Protein profiles of IZON fractions and pooled samples were assessed via SDS–PAGE using NuPAGE 4%-12% precast gels (Thermo Fisher Scientific, USA), stained with SimplyBlue™ SafeStain (Thermo Fisher Scientific, USA), and visualized using a Gel Doc imaging system (Bio-Rad, USA). A 10-180 kDa PageRuler protein ladder (Thermo Fisher Scientific, USA) was used as a molecular weight reference. A 20 µL aliquot of each sample, corresponding to 1.5 × 10^10^ LpEVs and 0.7 × 10^10^ LpEVs^BILE^, was loaded per lane.

#### Proteomics

Proteins were extracted in SDT buffer (4% SDS, 0.1 M DTT, 0.1 M Tris-HCl, pH 7.6) at 95 °C in a thermomixer (750 rpm; ThermoMixer C, Eppendorf, Germany) for 30 min (EVs) or 60 min (bacteria). After centrifugation (20,000 × *g*, 15 min, RT), 5 µg of total protein from each sample was processed using the filter-aided sample preparation method^[20]^ with sequencing-grade trypsin (0.5 µg; Sigma-Aldrich, USA). Resulting peptides were extracted with 2.5% formic acid (FA) in 50% acetonitrile (ACN), followed by extraction with 100% ACN, both solutions containing 0.001% polyethylene glycol^[21]^. Peptides were then concentrated using a SpeedVac concentrator (Thermo Fisher Scientific, USA).

Liquid chromatography–tandem mass spectrometry was performed using an UltiMate 3000 RSLCnano system (Thermo Fisher Scientific, USA) coupled to a timsTOF Pro mass spectrometer (Bruker Optics GmbH, Germany). Samples were desalted on a trap column (Acclaim PepMap 100 C18, 300-µm ID, 5-mm length, 5-µm particle size; Thermo Fisher Scientific, USA) and washed with 0.1% trifluoroacetic acid. Peptides were eluted in back-flush mode from the trap column onto an analytical column (Aurora C18, 75-µm ID, 250-mm length, 1.7-µm particle size; Ion Opticks, Australia) using a 60-min gradient (3%-42% mobile phase B; mobile phase A: 0.1% FA in water; mobile phase B: 0.1% FA in 80% ACN) at a flow rate of 150 nL/min, followed by a system wash with 80% mobile phase B. The trapping and analytical columns were equilibrated before sample injection into the sample loop. The analytical column was installed in the CaptiveSpray ion source (Bruker Optics GmbH, Germany) maintained at 50 °C, following the manufacturer’s instructions. The spray voltage and sheath gas were set to 1.5 kV and 1.0 L/min, respectively.

MSn data were acquired in data-independent acquisition (DIA) mode with a base m/z range of 100 to 1,700 and a 1/K0 range of 0.6 to 1.4 V·s·cm^-2^. The enclosed file, DIAparameters.txt, defines an m/z 400-1,000 precursor range with equal window sizes of 26 Th, using two steps for each PASEF scan and a cycle time of 100 ms locked to a 100% duty cycle. High-sensitivity detection mode was applied to measure the EV samples to compensate for the low protein digestion input. DIA data were processed in DIA-NN (version 1.8.1)^[22]^ in library-free mode against the cRAP database (http://www.thegpm.org/crap; 111 sequences, version 2018/11) and the UniProtKB protein database for *L. plantarum* NCIMB 8826 (https://www.uniprot.org/proteomes/UP000000432; version 2024/01; 3,087 protein sequences). Carbamidomethylation was set as a fixed modification, and trypsin/P was selected as the digestion enzyme, allowing one missed cleavage and peptide lengths of 7-30 amino acids. The false discovery rate (FDR) threshold was set to 1% at both the precursor and protein levels. MS1 and MS2 accuracies, as well as scan window parameters, were optimized based on initial test searches, using the median parameter values across all samples. Match-between-runs was enabled. Protein MaxLFQ intensities reported in the DIA-NN main output file were further processed using the Omics Workflows containerized environment (https://github.com/OmicsWorkflows; version 4.7.7a). The downstream data-processing workflow (available upon request) included (a) removal of low-quality precursors and contaminant protein groups, (b) normalization of precursor intensities using the loessF algorithm, (c) calculation and log2 transformation of protein group MaxLFQ intensities, and (d) differential expression analysis using the LIMMA statistical test. Proteins with an adjusted *P*-value ≤ 0.05 and a fold change > 2 were considered significantly altered.

The mass spectrometry proteomics data have been deposited in the ProteomeXchange Consortium via the PRIDE partner repository^[23]^ under the dataset identifier PXD060959.

Principal component analysis (PCA) was performed on proteomic data quantified as MaxLFQ intensities. Prior to analysis, features were filtered to retain the 500 most variable proteins based on standard deviation (SD) across samples. The resulting dataset was standardized (z-score scaling) to ensure equal contribution of all variables regardless of absolute abundance. PCA was then conducted on the scaled data to explore patterns of variance and sample clustering across experimental groups. Volcano plots were generated using VolcaNoseR (https://huygens.science.uva.nl/VolcaNoseR/)^[24]^, including proteins identified in at least two samples. Statistical analysis of raw proteomic data was performed using quantitative values processed with the LIMMA statistical package in R. A significance threshold of *P* ≤ 0.05 and an absolute fold-change cutoff of 2 were applied. Manhattan distance was used for ranking significant hits.

STRING analysis in multiple-protein mode was performed on significantly upregulated proteins (*P* ≤ 0.05 and a fold-change cutoff of ≤ -2 or ≥ 2) (LpEVs^BILE^ *vs.* LpEVs) and proteins uniquely present in LpEVs^BILE^ (904 proteins in total). Statistical significance was determined using a hypergeometric test (equivalent to Fisher’s exact test), with multiple testing correction applied using the Benjamini–Hochberg procedure to control the FDR. Enriched terms with an adjusted *P*-value (FDR) ≤ 0.05 were considered statistically significant. STRING default parameters were used. The *L. plantarum* WCFS1 protein database was used as the background reference.

#### FTIR spectroscopy

Changes in the metabolic fingerprints of *L. plantarum*, bile-exposed bacteria (Lp^BILE^), and their corresponding EVs (LpEVs and LpEVs^BILE^) were assessed via FTIR spectroscopy. Three independent cultures were prepared, from which bacterial cells and EVs were collected for matched analyses (three biological repeats). Bacterial cells were pelleted (3,000 × *g*, 20 min) and washed with 25 mM HEPES-N. In parallel, EVs from the same cultures were purified as described above. Aliquots (10 µL) of each sample were applied to silicon optical plates (Bruker Optics GmbH, Germany) and dried at 40 °C for 30 min. Spectra were recorded in transmission mode using an HTS-XT microplate adapter coupled to a Tensor 27 FTIR spectrometer (Bruker Optics GmbH, Germany) under the following conditions: spectral range of 4,000-500 cm^-1^, spectral resolution of 6 cm^-1^, and averaging of 32 interferograms with background subtraction for each spectrum.

Spectra were preprocessed by vector normalization and baseline correction^[25]^. Hierarchical cluster analysis (HCA) was performed on the full spectral range and on regions corresponding to fatty acids (3,020-2,800 cm^-1^), proteins (1,720-1,500 cm^-1^), and polysaccharides (1,200-900 cm^-1^).

### EV-TRACK

Experimental details have been submitted to the EV-TRACK knowledge base (EV-TRACK ID: EV250022)^[26]^.

### Statistical analysis

Statistical analyses were performed using GraphPad Prism version 9.5.1, unless otherwise specified. Comparisons between groups were conducted using one-sample *t*-tests, ordinary one-way ANOVA, Dunnett’s multiple-comparisons test with a single pooled variance, or two-way ANOVA, as appropriate. The statistical tests used are indicated in the corresponding figure legends. A *P*-value of *P* ≤ 0.05 was considered statistically significant.

## RESULTS

### Characterization and stability of LpEVs isolated from *L. plantarum* cultured in MRS-T

*L. plantarum* was cultured in MRS-T, and LpEVs were harvested from 5 L cultures at an OD600 of 1.5, corresponding to the mid-logarithmic growth phase. Following filtration to remove cells and debris, crude EVs were isolated via UC. Further purification was performed using SEC [Figure 1], resulting in eight fractions of 400 µL each (Supplementary Figure 2A and B shows a representative chromatogram). Typically, fractions 1-3 (PCV 1.1-1.3) were pooled, resulting in a final volume of approximately 1.2 mL of purified LpEVs per batch.

**Figure 2:**
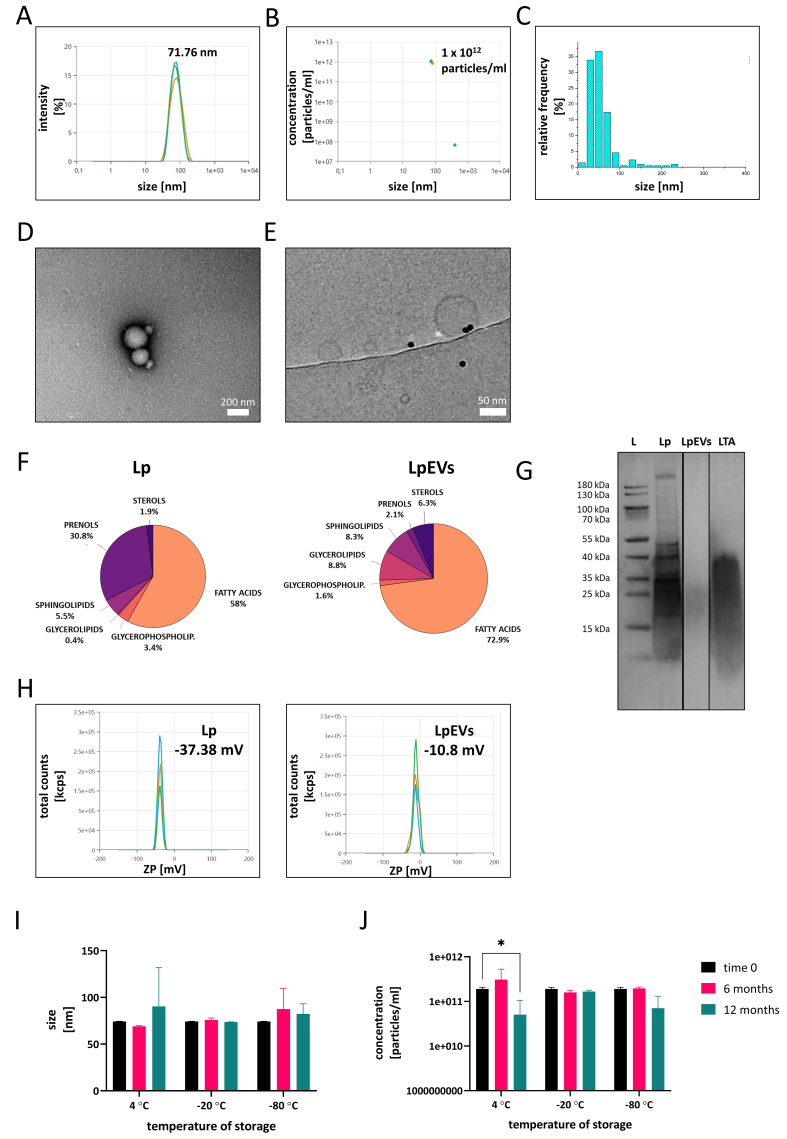
Characterization of LpEVs. (A) Representative measurements of particle size and concentration (B) of LpEVs obtained using a Zetasizer; (C) Size distribution of LpEVs determined using cryo-EM; (D) TEM and (E) cryo-EM visualization of LpEVs; (F) Surface lipid composition of intact *L. plantarum* and LpEVs analyzed via ToF-SIMS; (G) Western blot analysis of LTA in *L. plantarum* lysates and LpEVs (L, protein ladder; 10 µg of *L. plantarum* lysate, 1.5 × 10^10^ LpEVs, and 10 µg of commercial LTA were loaded per lane); (H) ZP measurements of *L. plantarum* and LpEVs in 1 mM HEPES buffer; Long-term stability of LpEVs stored in 25 mM HEPES-N buffer at 4, -20, and -80 °C showing changes in particle size (I) and concentration (J) over time. Data were analyzed using two-way ANOVA followed by Dunnett’s multiple-comparisons test. Significant differences are indicated as ^*^P ≤ 0.05. LpEVs: *L. plantarum* EVs; cryo-EM: cryogenic electron microscopy; TEM: transmission electron microscopy; *L. plantarum*: *Lactiplantibacillus plantarum*; ToF-SIMS: time-of-flight secondary ion mass spectrometry; LTA: lipoteichoic acid; ZP: zeta potential; HEPES: 4-(2-Hydroxyethyl)piperazine-1-ethanesulfonic acid sodium salt; HEPES-N: HEPES with 0.9% NaCl; ANOVA: analysis of variance.

Dynamic light scattering (DLS) analysis using a Zetasizer revealed a mean particle size of 73.9 nm (SD = 6.9 nm) and an average particle concentration of 1.64 × 10^12^ particles/mL (SD = 0.91 × 10^12^ particles/mL) across five independent batches (five biological replicates) [Supplementary Table 1]. The average total yield per 5 L culture was 1.97 × 10^12^ LpEVs (SD = 1.09 × 10^12^). Representative particle size and concentration data from one batch are shown in Figure 2A and B. Purification via SEC markedly reduced the protein and PGN content in the purified EV preparations compared with crude EVs obtained via UC alone [Supplementary Figure 2C and D]. To contextualize LpEV production relative to bacterial biomass, the colony-forming unit (CFU) count of the parent bacteria was determined in the same 5 L culture used for EV isolation. At an OD600 of 1.5, the culture contained 6.7 × 10^12^ CFUs and yielded 1.2 × 10^12^ LpEVs, corresponding to an approximate bacterium-to-EV ratio of 6:1.

Because the determination of EV size largely depends on the analytical method used^[8]^, cryo-EM was employed as a complementary technique to assess the size distribution of LpEVs [Figure 2C]. Compared with DLS measurements obtained using the Zetasizer, cryo-EM analysis showed that LpEVs predominantly ranged within 20-50 nm in diameter.

Transmission electron microscopy (TEM) [Figure 2D and Supplementary Figure 3] and cryo-EM [Figure 2E] were used to visualize the morphology of LpEVs and assess the presence of aggregates or bacterial debris. TEM images showed intact, spherical vesicles that frequently appeared in clusters [Figure 2D], as further confirmed by wide-field TEM views [Supplementary Figure 3]. Cryo-EM provided definitive confirmation of vesicle identity and revealed a well-defined bilayer membrane even in the smallest EVs [Figure 2E], underscoring their structural integrity.

Because MRS-T contains components derived from animal products, it was important to assess whether EVs or EV-like particles originating from the medium itself could confound the experimental results. To address this, MOCK-EVs were isolated from 5 L of uninoculated MRS-T and processed using the same protocol as for bacterial cultures. DLS analysis showed that MOCK-EVs were larger than LpEVs [Supplementary Figure 4A], with a concentration of 2.12 × 10^10^ particles/mL - approximately 100-fold lower than that observed in *L. plantarum* cultures [Supplementary Figure 4B]. TEM revealed only a few particles in MOCK-EV samples, exhibiting morphological features distinct from those of LpEVs [Supplementary Figure 4C]. No protein bands were detected when the maximum possible sample volume (20 µL) was loaded onto the SDS–PAGE gel [Supplementary Figure 4D]. Moreover, the protein content of the MOCK-EV samples, determined via both BCA and Bradford assays, was below the detection limit (data not shown). These findings confirm that EV-like particles present in MRS-T are negligible in both abundance and protein content and are therefore unlikely to interfere with the interpretation of LpEV-specific experimental results.

The surface lipid composition of *L. plantarum* and LpEVs was analyzed using the semi-quantitative ToF-SIMS method. The analysis revealed a decrease in prenols and an enrichment of fatty acids on the surface of LpEVs compared with the parent bacteria [Figure 2F]. Conversely, levels of glycerophospholipids, sphingolipids, and sterols showed only minor differences between the bacteria and their EVs. The presence of LTA, a key structural component of Gram-positive bacteria, was confirmed in both the parent cells and LpEVs via western blotting [Figure 2G]. ZP measurements performed in 1 mM HEPES buffer revealed distinct differences in surface charge between the bacteria and their EVs [Figure 2H]. The bacterial surface exhibited a strong negative charge of -37.38 mV (SD = 0.25 mV), whereas LpEVs displayed a considerably less negative charge of -10.8 mV (SD = 1.01 mV). These results indicate substantial differences in the surface properties of LpEVs and their parental cells, which may critically influence their stability, interactions, and biological functionality.

LpEVs were routinely stored in HEPES-N buffer at -20 °C. To evaluate their long-term stability under different conditions, we analyzed LpEV samples stored in HEPES-N at three temperatures: 4, -20, and -80 °C [Figure 2I and J]. At 4 °C, signs of instability became evident after 12 months, including significant fluctuations in particle concentration (*P* ≤ 0.05). Conversely, samples stored at -20 and -80 °C remained stable for up to one year. We further tested four alternative buffer formulations for their ability to preserve LpEVs: (i) PBS, (ii) PBS-A, (iii) PBS-HA, and (iv) HEPES-NA [Supplementary Figure 5]. At 4 °C, none of the buffers maintained LpEV stability beyond six months; complete degradation occurred, as no reliable measurements could be obtained (low data quality reported by the Zetasizer) [Supplementary Figure 5A]. Conversely, storage at -20 °C [Supplementary Figure 5B] and -80 °C [Supplementary Figure 5C] preserved vesicle integrity across all buffer conditions. Notably, buffers containing albumin led to an apparent increase in particle size, suggesting aggregation or interaction effects at 4 °C. Based on these results, we recommend storing LpEVs at -20 °C in 25 mM HEPES-N buffer as the optimal condition for maintaining vesicle integrity and concentration during long-term storage.

### LpEVs retain their size and numbers under variable pH, high salt and low SDS concentrations

**Figure 3:**
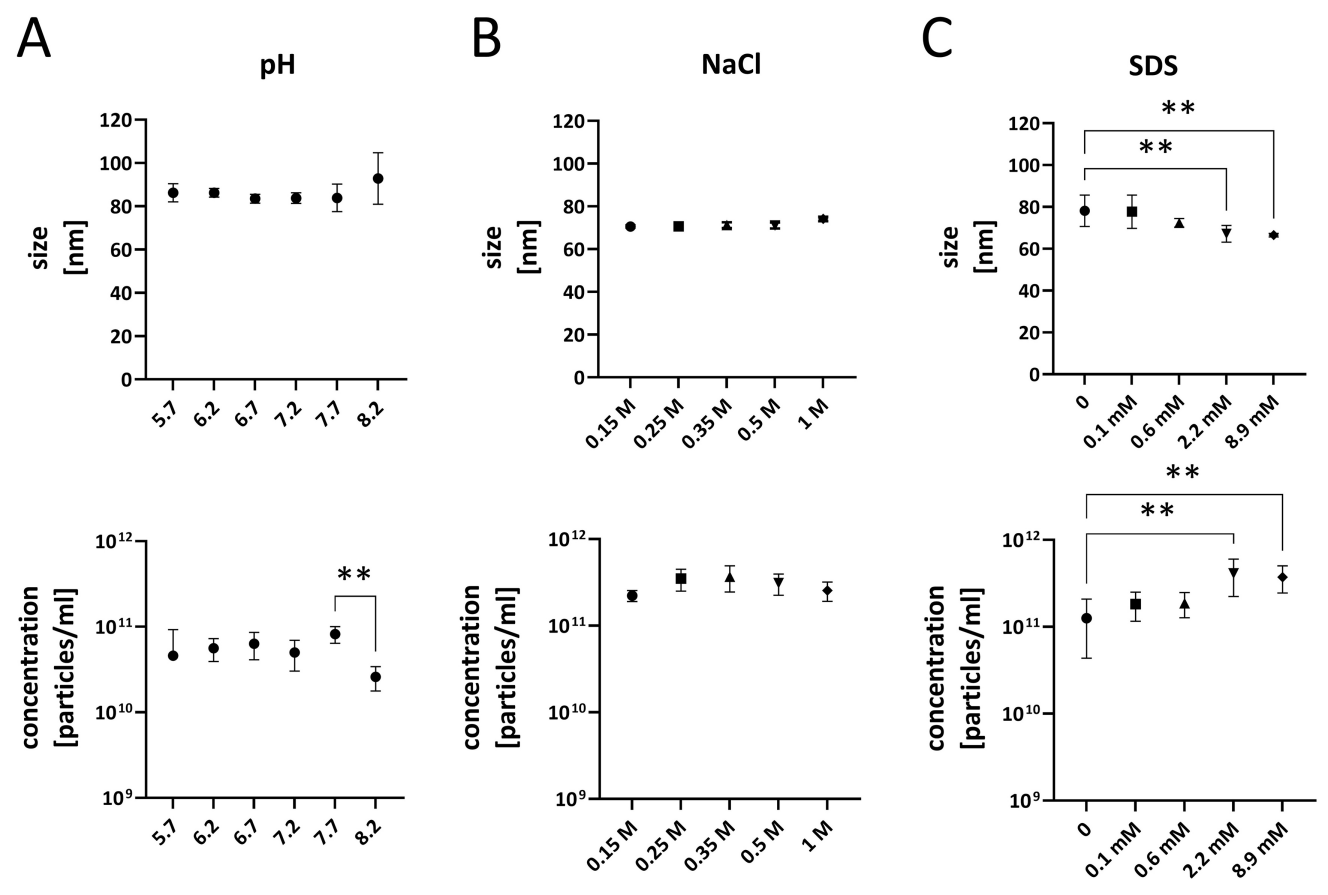
Influence of pH, salt, and detergent on the stability of LpEVs. Particle size and concentration of LpEVs were measured after exposure to different pH values (A), NaCl concentrations (B), and SDS concentrations (C) using a Zetasizer. Data were analyzed using one-way ANOVA followed by Dunnett’s multiple-comparisons test. Significant differences are indicated as ^**^P < 0.01. LpEVs: *L. plantarum* EVs; SDS: sodium dodecyl sulfate; ANOVA: analysis of variance.

Given the apparent physical robustness of LpEVs, we evaluated their stability under various environmental conditions that mimic those in the human body. Equivalent quantities of LpEVs were exposed to buffers of different compositions, and their particle size and concentration were measured using the Zetasizer. LpEVs remained stable in size and concentration across a pH range of 5.7-7.7. However, at pH values above 7.7, a significant decrease (*P* < 0.01) in LpEV concentration was observed, indicating sensitivity to alkaline conditions [Figure 3A]. LpEVs also demonstrated high tolerance to salt stress with respect to their size and concentration when incubated in NaCl concentrations up to 1 M [Figure 3B]. When exposed to the negatively charged detergent SDS, LpEVs maintained stable size and numbers at SDS concentrations up to 0.6 mM. At higher SDS concentrations, both particle size and concentration changed significantly (*P* < 0.01); however, LpEVs remained detectable even under these conditions, suggesting that complete vesicle degradation did not occur [Figure 3C].

### Growth of *L. plantarum* is significantly impaired by environmental stressors

**Figure 4:**
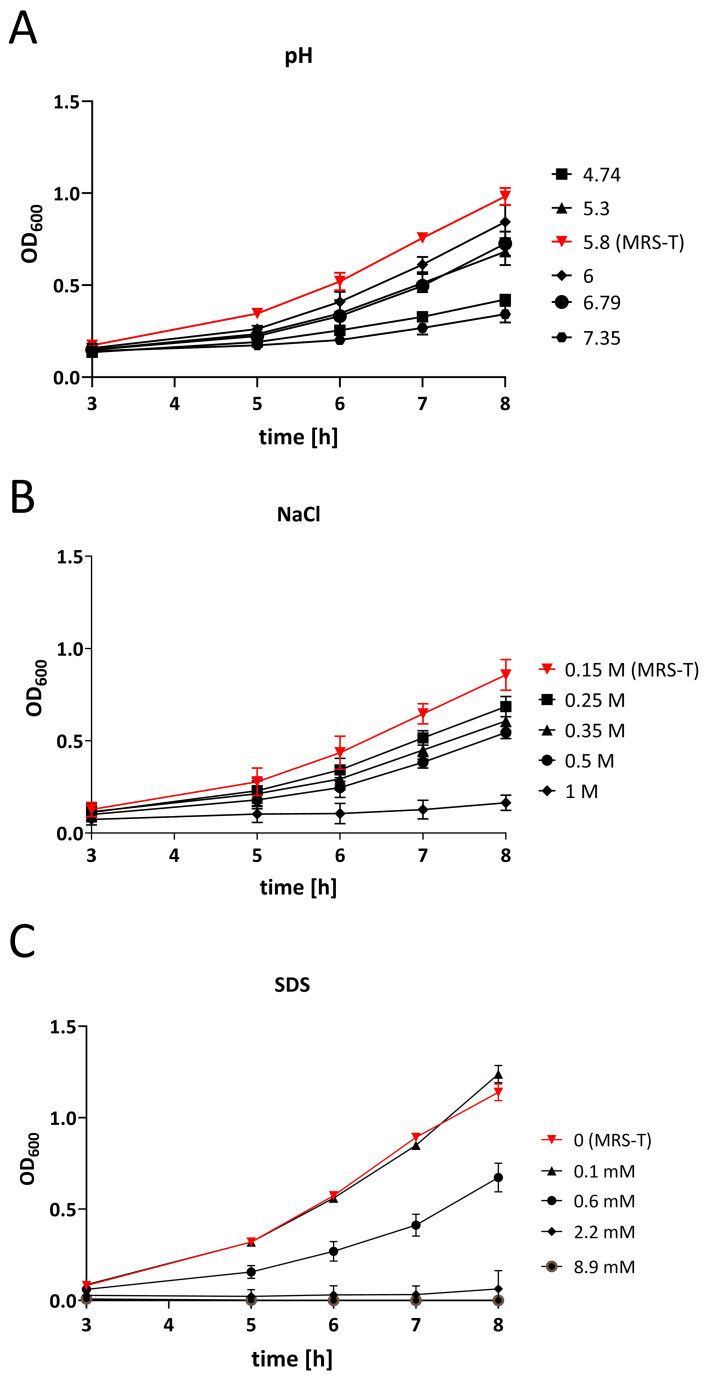
Influence of pH, salt, and detergent on *L. plantarum* growth. OD600 measurements of *L. plantarum* cultures grown in media (A) adjusted to pH values ranging within 4.74-7.35 (pH measured at the end of culture), (B) supplemented with NaCl concentrations of 0.15-1.0 M, and (C) supplemented with SDS concentrations of 0-8.9 mM. Experiment was performed in triplicate (three biological repeats) and three technical repeats. Data were analyzed using two-way ANOVA followed by Dunnett’s multiple-comparisons test (see statistical analysis in the text). *L. plantarum*: *Lactiplantibacillus plantarum*; SDS: sodium dodecyl sulfate; ANOVA: analysis of variance; MRS-T: De Man–Rogosa–Sharpe broth supplemented with Tween 80.

Given the demonstrated stability of LpEVs across a broad range of pH values, salt concentrations, and negatively charged detergents, we next examined how these environmental conditions affect the growth of the parent bacteria. *L. plantarum* was initially cultured in MRS-T, and overnight cultures were subsequently inoculated into fresh media modified to reflect varying pH, NaCl, and SDS concentrations [Figure 4]. Under standard conditions (pH 5.8), the bacteria exhibited robust growth. However, deviations from this pH significantly impaired bacterial proliferation, with significant reductions observed from 5 h post-inoculation (*P* < 0.0001, two-way ANOVA) [Figure 4A]. Similarly, increasing the NaCl concentration above 0.15 M led to dose-dependent growth inhibition, with significant differences detected at all tested concentrations from 6 h onward (*P* < 0.001, two-way ANOVA) [Figure 4B]. Exposure to SDS also markedly suppressed bacterial growth; even at 0.6 mM, SDS significantly reduced growth at 5, 6, 7, and 8 h post-inoculation (*P* < 0.0001, two-way ANOVA), with higher concentrations exerting progressively stronger inhibitory effects [Figure 4C].

### *L. plantarum* produces distinct EV populations in response to bile stress

As *L. plantarum* encounters bile salts during gastrointestinal transit, we investigated bile as a physiologically relevant stressor influencing bacterial growth, EV production, and EV-associated cargo. As shown above, environmental factors such as pH, salinity, and detergents affect bacterial growth dynamics. Extending this to bile, we examined the effects of increasing concentrations of bovine bile in MRS-T.

**Figure 5:**
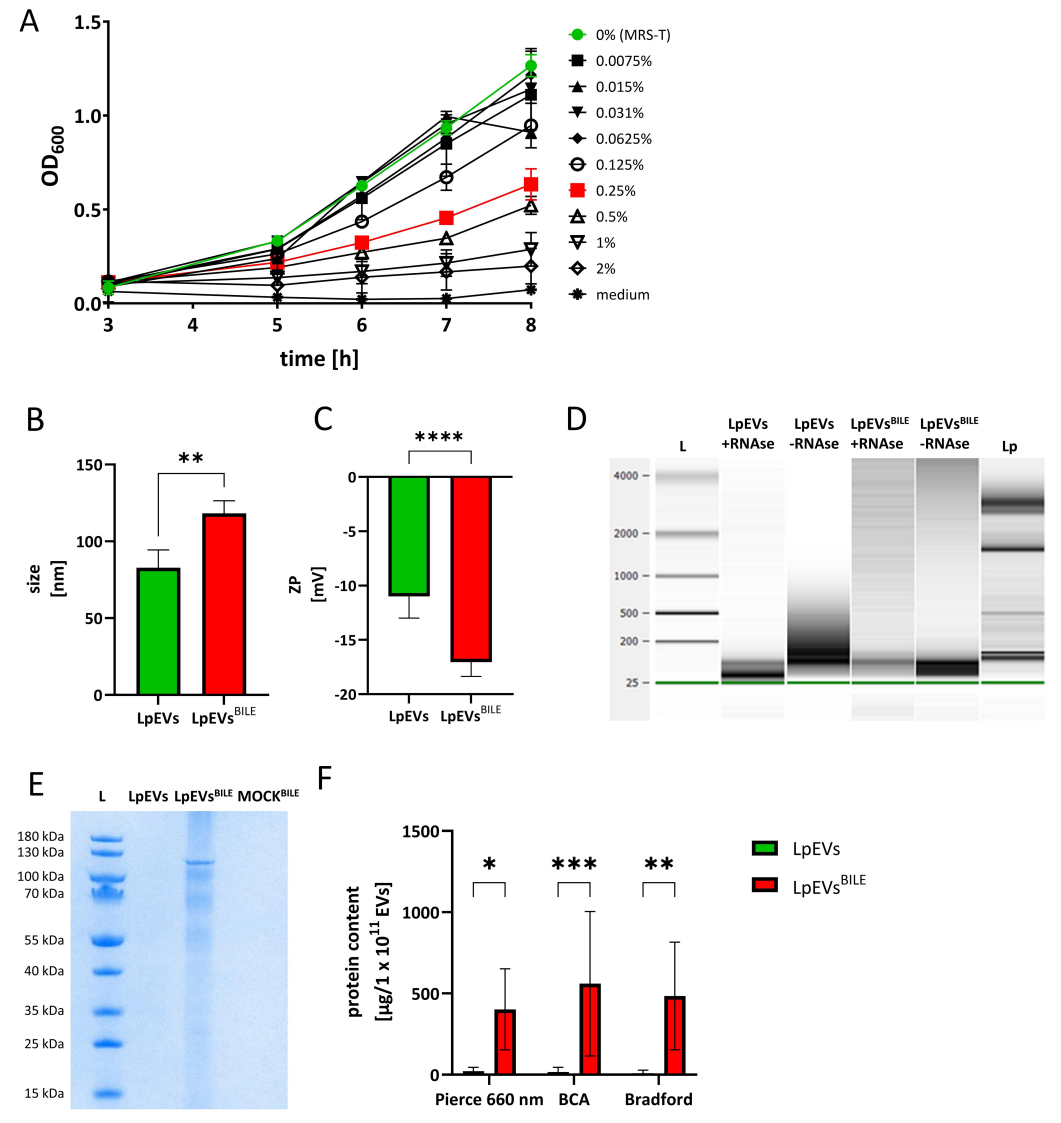
Influence of bile on the growth of *L. plantarum* and the characteristics of its EVs. (A) Growth curves of *L. plantarum* cultured in MRS-T supplemented with increasing concentrations of bovine bile (0%-2%). Three biological replicates; (B) Size distribution and (C) ZP measurements of LpEVs and LpEVs^BILE^ obtained using a Zetasizer. Three independent batches analyzed (biological replicates); (D) RNA content in untreated and RNase-treated EVs analyzed using a Bioanalyzer. L, RNA ladder; (E) SDS–PAGE of 1.5 × 10^10^ LpEVs and 0.7 × 10^10^ LpEVs^BILE^, was loaded per lane., and 20 µL of MOCK-EVs^BILE^. L, protein ladder; (F) Total protein content of LpEVs and LpEVs^BILE^ quantified using the Pierce 660 nm Protein Assay, BCA, and Bradford assays. Results are normalized to µg of protein per 1 × 10^11^ EVs and expressed as mean ± SD, data obtained from measurements of three independent EV batches (biological replicates). (B and C) were analyzed using unpaired two-tailed *t*-tests; (F) was analyzed using two-way ANOVA. Significance levels are indicated as ^*^P ≤ 0.05, ^**^P < 0.01, ^***^P < 0.001, and ^****^P < 0.0001. *L. plantarum*: *Lactiplantibacillus plantarum*; EVs: extracellular vesicles; MRS-T: De Man–Rogosa–Sharpe broth supplemented with Tween 80; ZP: zeta potential; LpEVs: *L. plantarum* EVs; SDS–PAGE: sodium dodecyl sulfate polyacrylamide gel electrophoresis; MOCK-EVs: EVs produced from medium without inoculation with bacteria; BCA: bicinchoninic acid; SD: standard deviation; ANOVA: analysis of variance.

Bile concentrations used in this study (0%-2%) were selected to span the physiological range encountered in the human small intestine, where luminal bile concentrations typically range from approximately 0.2% to 2% (w/v) under fed conditions^[27,28]^. A concentration of 0.25% was ultimately selected for detailed EV analysis as it represents a sublethal yet clearly stressful condition within this physiological range, causing approximately 50% inhibition of *L. plantarum* growth [Figure 5A] while still permitting sufficient bacterial growth and EV production for downstream characterization. EVs produced under bile stress (LpEVs^BILE^) were isolated and characterized using the same protocol as for EVs produced under standard conditions (LpEVs), with harvesting conducted at OD600 = 1.5. Although the total particle yield of LpEVs^BILE^ was comparable to that of LpEVs, DLS analysis revealed a significant increase (*P* < 0.01) in mean vesicle size, from 82.79 (SD = 11.63 nm) to 118.1 nm (SD = 8.35 nm) [Figure 5B]. Additionally, ZP measurements showed a more negative surface charge for LpEVs^BILE^ (-17.09 mV, SD = 1.33 mV) than for LpEVs (-10.99 mV, SD = 2 mV), suggesting alterations in membrane composition or structure [Figure 5C]. Analysis of vesicular RNA content using a Bioanalyzer revealed an enrichment of RNA in LpEVs^BILE^, increasing from 2.21 ng per 1 × 10^11^ EVs in LpEVs to 155.56 ng per 1 × 10^11^ EVs in LpEVs^BILE^. Following RNase treatment, both vesicle types retained sRNA fragments < 100 nucleotides, whereas untreated samples (particularly LpEVs) contained a broader range of RNA sizes, including longer fragments [Figure 5D]. No DNA was detected in either EV preparation using our assay (data not shown). However, this does not rule out the presence of low-abundance vesicle-associated DNA below our method’s detection limit.

To investigate the protein cargo of LpEVs and LpEVs^BILE^, we performed SDS–PAGE, quantitative protein assays using three independent methods, and subsequent proteomic analysis. SDS–PAGE revealed a distinct protein band at approximately 120 kDa in LpEVs^BILE^, whereas no visible bands were detected in LpEVs under identical loading conditions or in the MOCK-EVs^BILE^ sample [Figure 5E]. Given the typically low protein yield from bacterial EVs - particularly for LpEVs - this likely reflects protein concentrations below the detection limit of SDS–PAGE rather than a true absence of protein. Consistently, quantitative protein assays showed a significantly higher total protein content in LpEVs^BILE^ than in LpEVs (*P* ≤ 0.05 for Pierce 660 nm, *P* < 0.01 for Bradford, *P* < 0.001 for BCA) [Figure 5F], with no significant variation among the three methods used.

### FTIR spectroscopy revealed bile-induced spectral alterations in *L. plantarum* and its EVs

**Figure 6:**
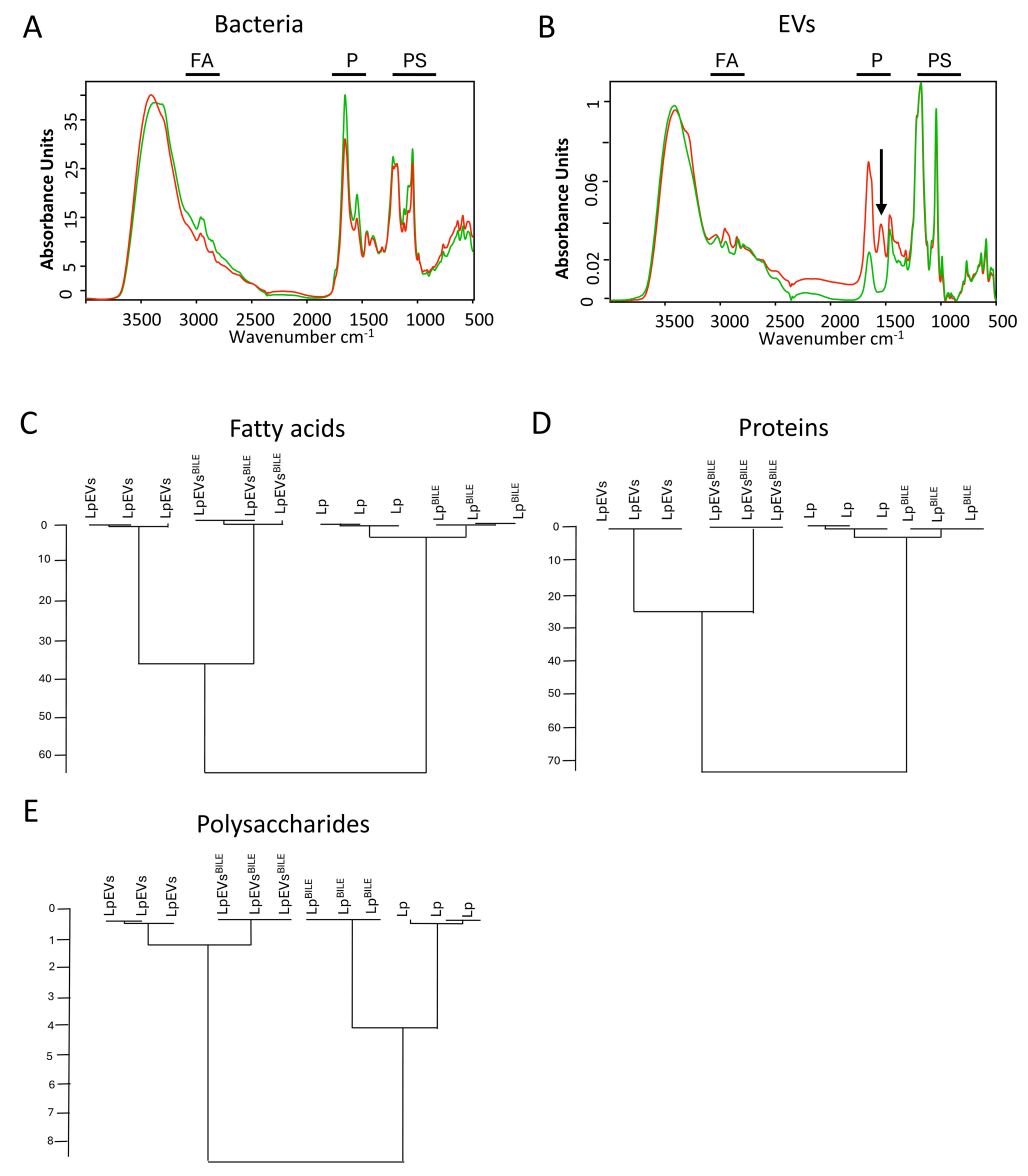
Metabolic fingerprints of *L. plantarum*, *L. plantarum*^BILE^, LpEVs, and LpEVs^BILE^ obtained via FTIR spectroscopy. FTIR spectral profiles of *L. plantarum*, *L. plantarum*^BILE^, LpEVs, and LpEVs^BILE^ were recorded and analyzed. Representative spectra for the parent bacteria (A) and EVs (B) are shown, with specific regions indicated: FA, fatty acids; P, proteins; PS, polysaccharides. HCA was performed on preprocessed and vector-normalized spectra using the Ward algorithm at repro level 30, based on spectral regions corresponding to fatty acids (C), proteins (D), and polysaccharides (E). Data represent three biological replicates; each measured in triplicate (technical replicates). *L. plantarum*: *Lactiplantibacillus plantarum*; LpEVs: *L. plantarum* EVs; FTIR: Fourier-transform infrared spectroscopy; EVs: extracellular vesicles; HCA: hierarchical cluster analysis.

FTIR spectroscopy was employed to investigate bile-induced metabolic changes in *L. plantarum* [Figure 6A] and its EVs [Figure 6B], building on previous studies demonstrating the utility of this technique for analyzing biochemical profiles of cells and vesicles^[25]^. Spectral data were recorded in the 4,000-500 cm^-1^ range and preprocessed before analysis. The effect of bile on LpEV spectra was evident across all three major spectral regions - proteins (1,720-1,500 cm^-1^), fatty acids (3,020-2,800 cm^-1^), and polysaccharides (1,200-900 cm^-1^) - but was most pronounced in the protein region [Figure 6B]. A distinct new peak appeared at 1,550 cm^-1^ in LpEVs^BILE^ that was absent in LpEVs (indicated by an arrow in Figure 6B). This wavelength is characteristic of chemical groups containing amide bonds, carboxylates, or nitro compounds. In contrast, spectral differences in the bacterial samples were primarily observed in the regions associated with fatty acids and polysaccharides [Figure 6A]. Baseline- and vector-normalized spectra from two additional biological replicates of bacteria and EVs are provided in the Supplementary Figure 6A and B. Additional control experiments (MOCKEV^BILE^ and MRST ± 0.244% bile) showed negligible FTIR spectral interference from bile acids or medium components [Supplementary Figure 7A and B], confirming that the observed LpEV^BILE^ signatures arise from vesicular material rather than bile- or medium-derived artifacts.

To further investigate the impact of bile on the spectral profiles of *L. plantarum* and LpEVs, HCA was performed. This analysis revealed clear clustering of samples according to the presence or absence of bile in the culture medium, whether based on the entire spectral range [Supplementary Figure 6C] or specific regions: fatty acids [Figure 6C], proteins [Figure 6D], and polysaccharides [Figure 6E]. Although the overall impact of bile on the spectral heterogeneity of *L. plantarum*/*L. plantarum*^BILE^ and LpEVs/LpEVs^BILE^ was comparable when analyzed across the full spectrum [Supplementary Figure 6C], region-specific differences became apparent [Figure 6C-E]. Spectral heterogeneity between LpEVs and LpEVs^BILE^ was more pronounced in the fatty acid and protein regions, whereas bacterial cells exhibited greater variability in the polysaccharide region.

### LpEVs^BILE^ show higher detection and relative abundance of BSH and catalytic enzymes

**Figure 7:**
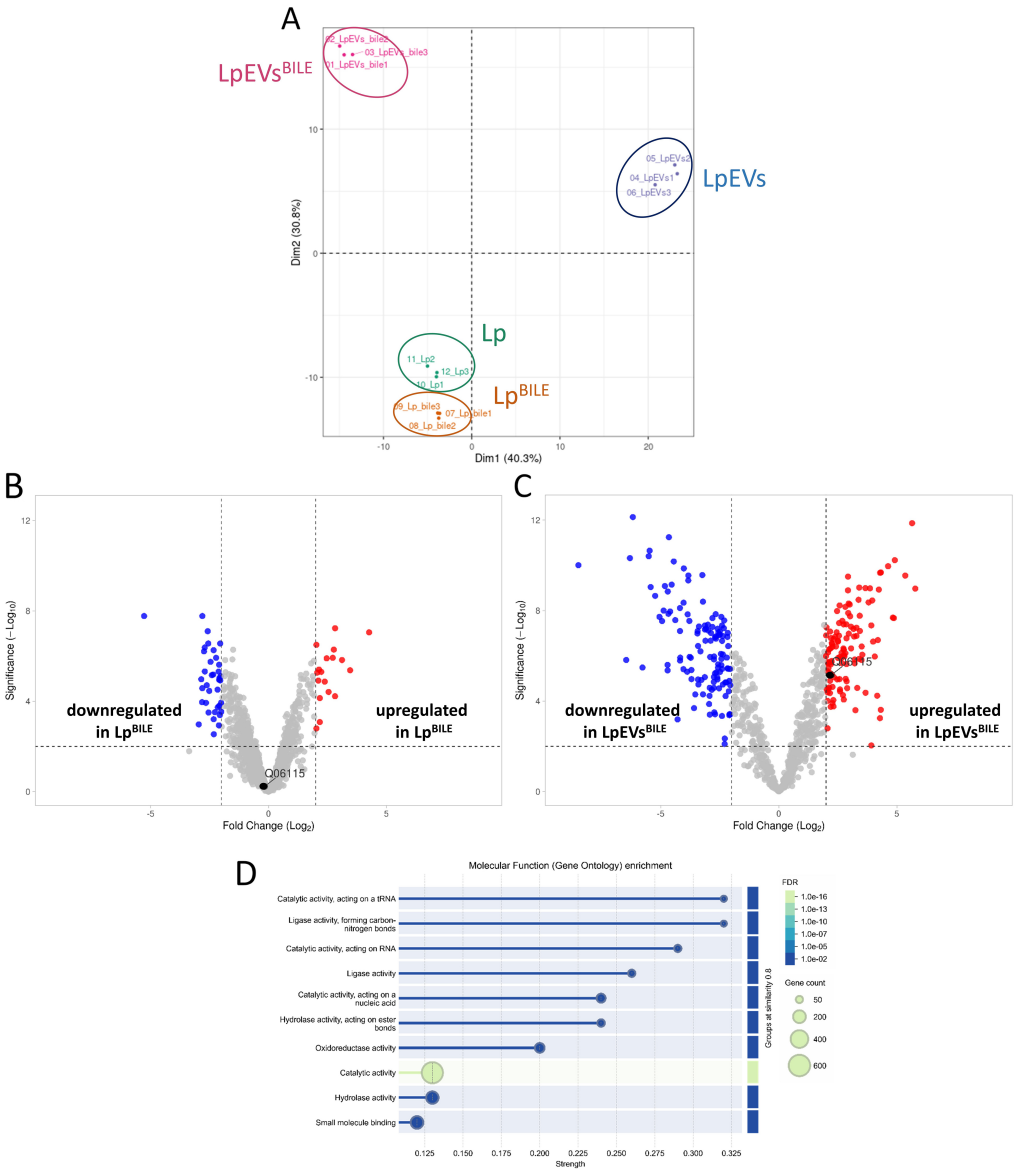
Proteomic analysis of *L. plantarum*, *L. plantarum*^BILE^, LpEVs, and LpEVs^BILE^. (A) PCA plot; (B) Volcano plot showing differential protein abundance between *L. plantarum* cultured without and with bile. BSH (Q06115) is highlighted with a black dot. Proteins with *P* ≤ 0.05 and a fold change ≥ 2 were considered significantly altered; (C) Volcano plot showing differences in the protein profiles of LpEVs^BILE^ compared with LpEVs. Proteins with *P* ≤ 0.05 and a fold change ≥ 2 were considered significantly altered. BSH is highlighted with a black dot; (D) Gene Ontology enrichment analysis of molecular functions in LpEVs^BILE^ compared with the *L. plantarum* reference proteome, performed using STRING. Enriched terms with adjusted *P* ≤ 0.05 (FDR) were considered statistically significant. *L. plantarum*: *Lactiplantibacillus plantarum*; LpEVs: *L. plantarum* EVs; PCA: principal component analysis; BSH: bile salt hydrolase; FDR: false discovery rate.

To investigate further qualitative differences in protein composition, we performed a comparative proteomic analysis of *L. plantarum* cultures grown with and without 0.25% bile, along with the corresponding EVs produced under these conditions. The proteomic workflow demonstrated excellent reproducibility across biological and technical replicates [Supplementary Figure 8A]. Consistent with the quantitative protein data [Figure 5F], LpEVs^BILE^ contained significantly higher total protein content and a greater number of identifiable proteins than LpEVs (1,533 proteins SD = 30.04 *vs.* 855.7 proteins SD = 53.91, *P* < 0.0001) [Supplementary Figure 8B]. For mass spectrometry, 250 ng of trypsin-digested peptide was analyzed for *L. plantarum*, *L. plantarum*^BILE^, and LpEVs^BILE^, whereas the maximum possible volume of LpEVs was used due to their limited protein content. PCA showed a well-structured separation of all sample groups [Figure 7A]. However, difference between Lp and Lp^BILE^ were subtle. LpEVs were separated from the other groups reflecting major compositional shift. Strong separation in PC1 and PC2 of LpEVs and LpEVs^BILE^ indicates that bile alters the EV protein composition. In bacterial cells, a total of 1,871 proteins were identified in both *L. plantarum* and *L. plantarum*^BILE^, with only 56 showing altered abundance [Figure 7B]. Among these, BSH, a key enzyme mediating bile acid deconjugation^[29]^, showed no significant change (*P* ≤ 0.05, fold-change cutoff of ≤ -2 or ≥ 2). Seventy-one proteins were unique to *L. plantarum*, whereas 22 were detected exclusively in *L. plantarum*^BILE^. In contrast, the EV proteome exhibited substantially greater variation. Across LpEVs and LpEVs^BILE^, 1,627 proteins were identified, with 101 showing significant differential abundance (*P* ≤ 0.05 and a fold-change cutoff of ≤ -2 or ≥ 2) [Figure 7C]. Notably, BSH was significantly upregulated in LpEVs^BILE^ compared with LpEVs. Furthermore, 82 proteins were unique to LpEVs, whereas 784 were detected exclusively in LpEVs^BILE^, indicating that bile stress induces a pronounced shift in EV cargo composition.

To investigate the identity of the prominent protein band observed exclusively in LpEVs^BILE^ by SDS-PAGE [Figure 5E], we re-examined the proteomic dataset for proteins within the corresponding molecular weight range of 105 to 125 kDa. Several proteins were detected exclusively in LpEVs^BILE^ and were absent from control LpEVs, suggesting that their incorporation into EVs is induced by bacterial exposure to bile rather than reflecting constitutive EV cargo. These include DNA polymerase III subunit alpha, carbamoyl phosphate synthase pyrimidine-specific large chain, a hypothetical membrane protein, cell surface protein (CscB family), Type I restriction enzyme endonuclease subunit (R protein), Sigma54 activator (mannose PTS operon regulator), 3′-5′ exonuclease DinG, and DNA translocase FtsK. These proteins are involved in core cellular processes including DNA maintenance, gene regulation, and metabolism/transport, suggesting that bile stress may trigger selective packaging of functionally significant cargo into LpEVs. While these candidates represent plausible identities for the band observed in bile-exposed samples, definitive assignment would require targeted mass spectrometric analysis of the excised gel band, which was beyond the scope of the current study.

Protein–protein interaction network and functional enrichment analyses performed using STRING revealed significant enrichment of proteins associated with molecular functions such as catalytic, ligase, hydrolase, and oxidoreductase activities [Figure 7D]. Enriched terms with adjusted *P* ≤ 0.05 (FDR) were considered statistically significant. Biological processes significantly enriched in LpEVs and LpEVs^BILE^ included the metabolism of pyridine-containing compounds, tRNA and RNA processing, noncoding RNA processing, and vitamin metabolic pathways [Supplementary Table 2]. Compared with the bacterial proteome background, LpEVs^BILE^ were predominantly enriched in cytosolic and cytoplasmic proteins [Supplementary Table 2]. Keyword-based annotation further revealed enrichment in flavoproteins, tRNA processing, ligases, oxidoreductases, transcription-associated proteins, and cytoplasmic proteins [Supplementary Table 2]. Of the 30 flavoproteins identified in *L. plantarum*, 24 were also detected in LpEVs^BILE^.

## DISCUSSION

Probiotics are live microorganisms that confer health benefits to the host when administered in adequate amounts^[30]^. These beneficial microbes support various physiological functions, both directly and indirectly, including protection against pathogens and attenuation of inflammation^[31]^. To exert these effects, probiotics must survive transit through the gastrointestinal tract, specifically by withstanding gastric acid and bile salts, and reach the intestine in sufficient numbers^[32]^. Previous studies have demonstrated that *L. plantarum* exhibits remarkable resilience under harsh conditions^[33]^. This resilience enables it to persist within the host environment, exerting both local immunomodulatory effects at mucosal surfaces and systemic benefits^[34,35]^. More recently, bacterial EVs have been recognized as key mediators in this process, providing a cell-free mechanism through which probiotics enhance host–microbe communication and contribute to environmental resilience^[36]^.

In this study, we provide new insights into the production, purification, characterization, and environmental adaptability of EVs from *L. plantarum* NCIMB 8826, originally isolated from human saliva. Through differential UC combined with SEC, and following the MISEV guidelines^[15]^, we obtained highly purified LpEVs with consistent physicochemical properties across multiple batches. The ability to produce EVs appears to be a conserved trait among *L. plantarum* strains, as isolates from fermented tea^[37]^, various foods^[38]^, and kimchi^[39,40]^ have also been reported to release EVs, highlighting the ecological versatility and potential functional relevance of EV production.

Our results also underscore the critical importance of the purification strategy. SEC substantially reduced the contents of co-isolated contaminants such as PGN and nonvesicular proteins, consistent with a previous report^[38]^. PGN, a key component of bacterial cell walls, is essential for maintaining structural integrity and resisting internal turgor pressure^[41]^. Although PGN was detected in the crude LpEV preparation, as also observed by Morishita *et al.*^[42]^, it was largely removed via SEC, supporting the interpretation that PGN represents a co-purified contaminant rather than a genuine EV cargo. This finding aligns with our previous observations in *E. coli* O83, where extracellular components were shown to co-purify with EVs and potentially confound functional analyses^[8]^. Because crude and purified EVs may exert distinct biological effects due to such contaminants, implementing rigorous and standardized EV isolation protocols is essential to enable meaningful comparisons across studies. Moreover, this raises an important question: do highly purified EVs truly reflect their physiological counterparts within the mammalian host? *In vivo*, EVs are likely to acquire a biomolecular corona - a layer of loosely or tightly associated host and environmental molecules - that can modulate their biological activity^[43]^. Although this phenomenon is well documented for mammalian EVs^[44,45]^, the existence and functional relevance of a corona on bacterial EVs remain largely unexplored. Addressing this knowledge gap will be crucial for balancing methodological stringency with biological relevance, ensuring that functional studies capture the true complexity of EV behavior within the host environment.

Although optimizing isolation techniques is essential for obtaining high-purity EVs, they are not the sole determinants of EV composition. Biological and environmental factors - including temperature^[11]^, growth medium composition^[46,47]^, pH^[48]^, culture duration^[49,50]^, and strain-specific characteristics^[51]^ - can independently influence EV yield, physicochemical properties, and immunomodulatory potential.

The biophysical properties of EVs also have functional significance. ZP analysis revealed that LpEVs possessed a lower surface charge than their parent cells, consistent with previous findings by Rogers *et al.*^[17]^, who reported that EVs often differ in surface charge from their parental bacteria. Such differences reflect variations in membrane composition and may influence vesicle stability, host interactions, and environmental adaptability.

Bacterial EVs also show great potential as vaccine platforms^[52]^. The clinical success of Bexsero - a licensed vaccine based on *Neisseria meningitidis* EVs - demonstrates the feasibility of translating bacterial EVs into effective immunotherapies^[53]^. However, to advance EV-based vaccines from bench to bedside, it is crucial to ensure their stability during storage and their resilience under physiologically relevant conditions^[19]^. LpEVs maintained size and concentration under several short-term environmental stress conditions, while long-term storage stability was best preserved at -20 °C in HEPES-N buffer; storage at 4 °C was associated with instability over extended periods. It should be noted that whether the lipid composition, protein cargo, or functional properties of LpEVs are maintained under these conditions was not assessed and represents a limitation of the current study.

Bacterial EVs are increasingly recognized as universal mediators of microbial and interspecies communication^[35,36]^. Owing to their small size and structural robustness, they can translocate from the gut into the portal circulation and deliver molecular cargo to peripheral sites^[37,38]^. Among these cargo components, RNA has emerged as a key determinant of functional relevance. In our study, LpEVs derived from both bile-treated and untreated cultures contained diverse RNA populations, including sRNAs protected from RNase digestion. Supporting their functional potential, Yu *et al.* demonstrated that sRNAs in EVs from the same *L. plantarum* strain can mediate interspecies signaling, with sRNA71 shown to suppress gene expression in human cells^[54]^. Interestingly, we observed an increased total RNA yield in LpEVs following bile exposure, suggesting that environmental conditions influence vesicular RNA content, possibly through a selective packaging mechanism. This observation aligns with previous findings in *Staphylococcus aureus*, wherein vesicular RNA profiles varied with growth phase and antibiotic stress^[55]^. Whether bile-induced sRNAs differ functionally from those in untreated EVs remains to be investigated.

Bile, a natural physiological stressor, regulates intestinal microbial populations by inhibiting excessive bacterial growth, thereby driving gut bacteria to evolve adaptive survival mechanisms^[56]^. Species such as *Lactobacillus* and *Bifidobacterium* respond to bile exposure by reinforcing their S-layer and increasing the production of polysaccharides, bile-responsive transporters, and enzymes such as BSH, which deconjugates bile acids. Beliakoff *et al.* demonstrated that bile modulates gene transcription in *Lactobacillus johnsonii*, particularly affecting genes involved in transport, biosynthesis, and cell wall processes during the late exponential phase^[47]^. In our study, which focused on *L. plantarum* during the logarithmic growth phase and on proteins regulated post-transcriptionally, we observed no substantial changes in the overall bacterial protein content in response to bile exposure. Neither proteomic analysis nor FTIR spectral profiling revealed significant alterations in protein expression or bacterial biochemical fingerprints. These findings are consistent with those of Koskenniemi *et al.*, who reported only minor proteomic changes after 60 min of bile exposure, suggesting that the activation of adaptive mechanisms may require a longer period to manifest^[57]^.

In our study, *L. plantarum* produced significantly larger EVs in response to bile exposure, without a corresponding increase in vesicle number. This contrasts with previous observations in *L. johnsonii* N6.2, wherein bile treatment increased both EV size and abundance^[47]^. The discrepancy in vesiculation patterns may reflect species-specific differences in membrane composition, EV biogenesis pathways, or stress-response mechanisms activated by bile. One possible explanation is that *L. plantarum* exerts stricter control over membrane remodeling under bile stress. Alternatively, bile may promote vesicle enlargement through membrane fusion or altered budding dynamics, independent of vesicle yield. Overall, these findings underscore the influence of strain-specific physiological traits on EV production under environmental stress.

Interestingly, exposure to bile also altered the surface charge of LpEVs, as indicated by a marked reduction in ZP. A more negatively charged surface could influence vesicle stability, colloidal behavior, and interactions with host cells or mucosal surfaces. Similar effects have been reported in *Bifidobacterium* species, wherein the steroid moieties of bile salts interact with hydrophobic domains on the bacterial surface^[58]^. It is plausible that analogous interactions occur at the LpEV membrane, thereby modulating its surface charge and molecular composition. Supporting this hypothesis, LpEVs^BILE^ consistently exhibited a yellow coloration, in contrast to the colorless appearance of control vesicles, suggesting direct incorporation or surface association of bile components (data not shown). In summary, given the presence of bile salts, and of Tween 80 in the growth medium, matrix contributions to the size and charge changes cannot be excluded.

FTIR spectroscopy provides a powerful approach for detecting metabolic and compositional changes in bacterial EVs in response to environmental stressors. Although FTIR spectroscopy has previously been applied to fingerprint *Bacillus cereus* and its EVs^[25]^, our study extends its utility by demonstrating its sensitivity to bile-induced alterations. Specifically, FTIR spectroscopy revealed significant alterations in spectral regions corresponding to proteins and fatty acids. Of particular interest was the appearance of a peak at 1,550 cm^-1^ in LpEVs^BILE^ compared with control EVs, consistent with the characteristic infrared absorbance of the isoalloxazine ring in flavin cofactors^[59]^. Given the established role of flavoproteins in bile metabolism^[60]^, this spectral feature may indicate the selective incorporation of flavin-dependent enzymes into EVs in response to bile exposure.

Further proteomic analysis revealed clear separation of analyzed groups (PCA) and significant compositional changes in LpEVs^BILE^: 101 proteins were differentially abundant, whereas 784 were uniquely detected compared with control LpEVs. Notably, enzymes with catalytic, hydrolytic, and ligase activities - including BSH - were enriched, suggesting effects on lipid, polysaccharide, and bile acid metabolism. This functional signature may reflect adaptive responses to bile stress or suggest regulated cargo selection, whereby *L. plantarum* selectively packages proteins into EVs under specific environmental conditions.

This study has several limitations. Bile exposure was modeled *in vitro* using a single bile source and a fixed sublethal concentration (0.25%), whereas *in vivo* bile composition and levels fluctuate in response to diet and host physiology. EVs were collected at a single growth phase, so potential temporal or growth phase–dependent dynamics of bile-induced EV remodeling were not captured. The functional impact of bile-stressed LpEVs on host cells remains inferential, as our conclusions are based on compositional (proteomic and FTIR) changes rather than on direct assays of epithelial or immune responses.

A key limitation of our proteomic analysis is the asymmetry in total protein yield between LpEVs and LpEVs^BILE^ samples, which reduces detection sensitivity for low-abundance proteins in the control vesicles and complicates the interpretation of proteins found exclusively under bile exposure conditions. Although we applied stringent identification filters, requiring a minimum of two unique peptides and detection in at least two biological replicates, and used normalized label-free intensities to account for differences in protein input, we cannot fully exclude the possibility that some proteins appearing unique to LpEVs^BILE^ reflect detection limits in the control condition rather than true absence. Accordingly, conclusions regarding proteins detected exclusively in bile-exposed EVs are based on qualitative presence/absence observations rather than formal statistical testing and should be interpreted with appropriate caution.

Nonetheless, our findingsalign with those of Stentz *et al.*, who demonstrated that *B. thetaiotaomicron* produces EVs enriched in proteins linked to bile acid metabolism *in vivo*^[61]^. Such stress-responsive sorting mechanisms may promote bacterial survival or modulate host physiology through EVs.

The FTIR spectra of *L. plantarum* also showed more subtle changes, primarily in the polysaccharide region following bile exposure, supporting a previously reported finding that stress can alter polysaccharide content^[62]^. Proteomic analysis further corroborated this stress-adaptive response. Notably, the transcriptional repressor LsrR, which negatively regulates polysaccharide biosynthesis^[63]^, was consistently detected in LpEVs^BILE^ but was absent in control LpEVs. This selective packaging suggests that *L. plantarum* may employ EVs to export regulatory proteins such as LsrR, potentially modulating gene expression and contributing to stress adaptation. Similar phenomena have been described in other biological systems, where bacterial EVs transfer enzymes or transcriptional regulators that alter carbohydrate utilization, stress responses, or virulence in neighboring cells. In Gram-negative bacteria, outer membrane vesicles (OMVs) have been shown to carry periplasmic enzymes and transcriptional regulators that reshape nutrient acquisition, redox balance, and stress adaptation in recipient cells^[62,63]^. In Gram-positive systems more closely related to *L. plantarum*, EVs from lactic acid bacteria and staphylococci have been reported to deliver cargo that modulates cell wall metabolism and stress tolerance in neighboring cells^[13,64]^. Collectively, these precedents support the hypothesis that EV-associated regulatory proteins, including quorum sensing regulators such as LsrR, may participate in intercellular metabolic modulation, potentially influencing neighboring microbial communities or host cells in the gut environment. Although our study does not directly demonstrate functional transfer, this hypothesis is biologically plausible and warrants targeted experimental testing in future work.

In parallel, bile exposure upregulated several stress-response regulators in both bacterial cells and EVs, including Gls24 family homologs Asp1 and Asp2, and the small heat shock protein Hsp3. These proteins are associated with membrane stabilization, protein folding, and general stress resilience, underscoring the coordinated physiological adaptation of *L. plantarum* to bile challenge. Overall, this study highlights the value of integrating FTIR spectroscopy–based metabolic profiling with proteomic analysis to elucidate bile-induced biochemical dynamics in both bacterial cells and their EVs.

To sum up, under bile stress, LpEVs showed increased particle size and protein load, together with a selective enrichment of proteins involved in bile metabolism and stress adaptation, including BSH, Gls24 family proteins (Asp1, Asp2), small heat shock protein Hsp3, and the transcriptional repressor LsrR. In parallel, FTIR analysis revealed bile-induced changes in lipid and polysaccharide-associated bands, indicating remodeling of the vesicle membrane and surface components. These alterations suggest that bile not only enhances EV biogenesis but also redirects their cargo toward functions that support survival in the intestinal environment: BSH-enriched vesicles may contribute to local bile detoxification and cholesterol metabolism; stress-response proteins may help stabilize membranes and proteins in producer and neighboring cells; and export of LsrR, a negative regulator of polysaccharide biosynthesis, could influence cell-wall remodeling or polysaccharide production at the community level. Together, these data support a model in which bile stress drives a shift in LpEVs from “housekeeping” cargo toward a stress-adapted, bile-oriented vesicle phenotype, with potential consequences for microbial fitness and host–microbe interactions in the small intestine.

## DECLARATIONS

### Availability of data and materials

The authors have deposited the following datasets in open repositories: ProteomeXchange Consortium via the PRIDE partner repository (dataset identifier PXD060959) and RODBUK Jagiellonian University in Kraków https://uj.rodbuk.pl/dataset.xhtml?persistentId=doi:10.57903/UJ/GLBXEG; DOI: 10.57903/UJ/GLBXEG). Additional data are available from the corresponding author upon reasonable request.

### AI and AI-assisted tools statement

During the preparation of this manuscript, the AI tool OpenAI GPT (version 5, released 2025-08-07) was used solely for language editing. The tool did not influence the study design, data collection, analysis, interpretation, or the scientific content of the work. All authors take full responsibility for the accuracy, integrity, and final content of the manuscript.

### Copyright

© The Author(s) 2026.
