# Supplementary material for: Extracellular vesicles as indicators of environmental stress response in Lactiplantibacillus plantarum
Source: Extracell Vesicles Circ Nucl Acids. 2026 May 29;7(2):786–812. doi: 10.20517/evcna.2026.26 (PMC13367151; doi:10.20517/evcna.2026.26)
Supplement: Supplementary file 1 [file evcna-7-2-786-SupplementaryMaterials.pdf]

Supplementary Materials

Extracellular vesicles as indicators of environmental stress response in *Lactiplantibacillus plantarum*

Agnieszka Razim[1,2], Astrid Laimer-Digruber[1,3], Anna M. Schmid[1], Tanja V. Edelbacher[3], Magdalena E. Paschall[1], Tamara Weinmayer[1], Michael Thaler[1], Rim Kebreab[1], Magdalena E. Skalska[4], Paweł Migdał[2], Mattia Morandi[5], Catherine Daniel[6], Dagmar Srutkova[7], Martin Schwarzer[7], Jiri Hrdy[8], Monika Ehling-Schulz[3], Sabina Górska[2], Aleksandra Inic-Kanada[1], Ursula Wiedermann[1], Irma Schabussova[1]

[1]Institute of Specific Prophylaxis and Tropical Medicine, Center for Pathophysiology, Infectiology and Immunology, Medical University of Vienna, Vienna 1090, Austria.

[2]Hirszfeld Institute of Immunology and Experimental Therapy, Polish Academy of Sciences, Wrocław 53-114, Poland.

[3]Institute of Microbiology, Centre for Pathobiology, Department of Biological Sciences and Pathobiology, University of Veterinary Medicine, Vienna 1210, Austria.

[4]Medical Physics Department, Marian Smoluchowski Institute of Physics, Jagiellonian University, Kraków 30-348, Poland.

[5]Institute of Organic Chemistry and Biochemistry, Czech Academy of Sciences, Prague 160 00, Czech Republic.

[6]Univ. Lille, CNRS, Inserm, CHU Lille, Institut Pasteur Lille, U1019 - UMR 9017 - CIIL - Center for Infection and Immunity of Lille, Lille F-59000, France.

[7]Laboratory of Gnotobiology, Institute of Microbiology, Czech Academy of Sciences, Nový Hrádek 549 22, Czech Republic.

[8]Institute of Clinical Immunology and Allergology, First Faculty of Medicine, Charles University and General University Hospital in Prague, Prague 121 11, Czech Republic.

Correspondence to: Dr. Agnieszka Razim, Hirszfeld Institute of Immunology and Experimental Therapy, Polish Academy of Sciences, Wrocław 53-114, Poland. E-mail: agnieszka.razim@hirszfeld.pl; Dr. Irma Schabussova, Institute of Specific Prophylaxis and Tropical Medicine, Center for Pathophysiology, Infectiology and Immunology, Medical University of Vienna, Vienna 1090, Austria. E-mail: irma.schabussova@meduniwien.ac.at

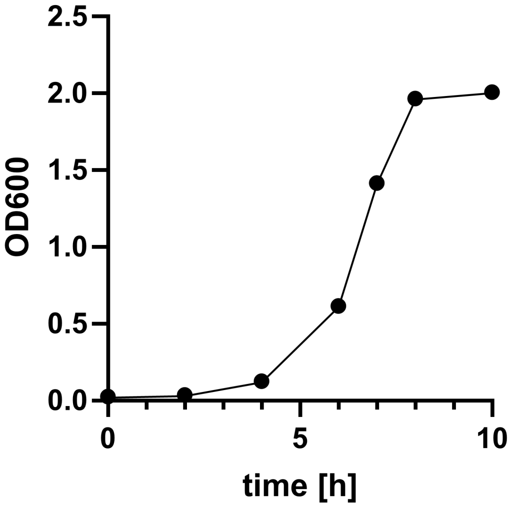

Supplementary Figure 1. *L.plantarum* growth curve. Cultures were grown in MRS broth supplemented with Tween 80 (MRS-T) in closed bottles without shaking at 37°C. The culture was inoculated with an overnight starter culture at a 1:200 ratio and OD600 was measured at 0, 2, 4, 6, 7, 8, and 10 h timepoints.

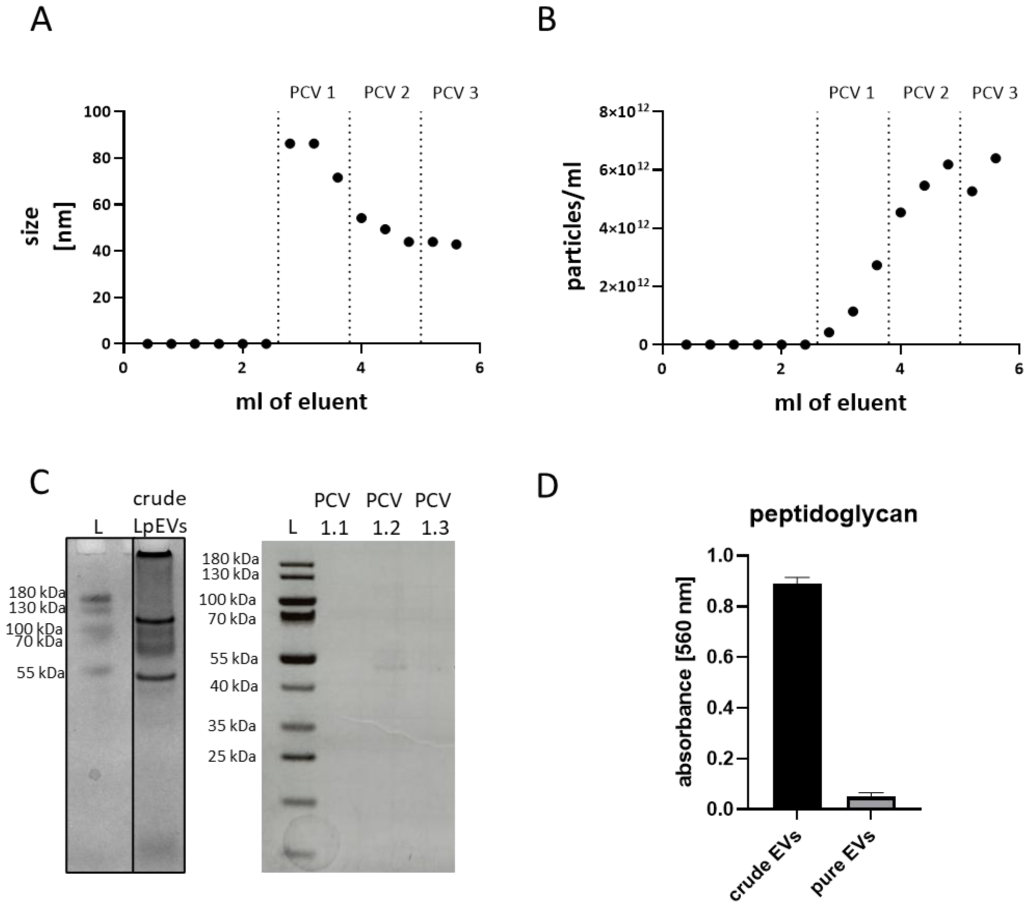

Supplementary Figure 2. Characteristics of LpEV fractions after SEC. Each dot represents one fraction. (A) Representative chromatogram showing particle size in the isolated fractions. (B) Representative chromatogram showing particle concentration in the isolated fractions. PCV, purified collection volume. (C) SDS–PAGE analysis of crude LpEVs (8×10^9 particles/well) and purified LpEVs (PCV 1.1 = 0.4×10^9 particles/well, PCV 1.2 = 7.6×10^9 particles/well, PCV 1.3 = 1.24×10^9 particles/well). (D) PGN measurement in 2.5×10^{10} crude and purified LpEVs.

Supplementary Table 1. Batch-to-batch variability in the isolation and purification of LpEVs

Batch number	Average particle size [nm]	Average particle concentration [particles/mL]
1	69.11	1.5×10^{12}
2	83.74	2×10^{12}
3	78.09	0.7×10^{12}
4	66.8	3×10^{12}
5	71.76	1×10^{12}
**Mean**	**73.9 ± 6.9**	**$1.64 \times 10^{12} \pm 0.91 \times 10^{12}$**

Data were collected from five independent batches, with bacterial cultures initiated from separate overnight starters.

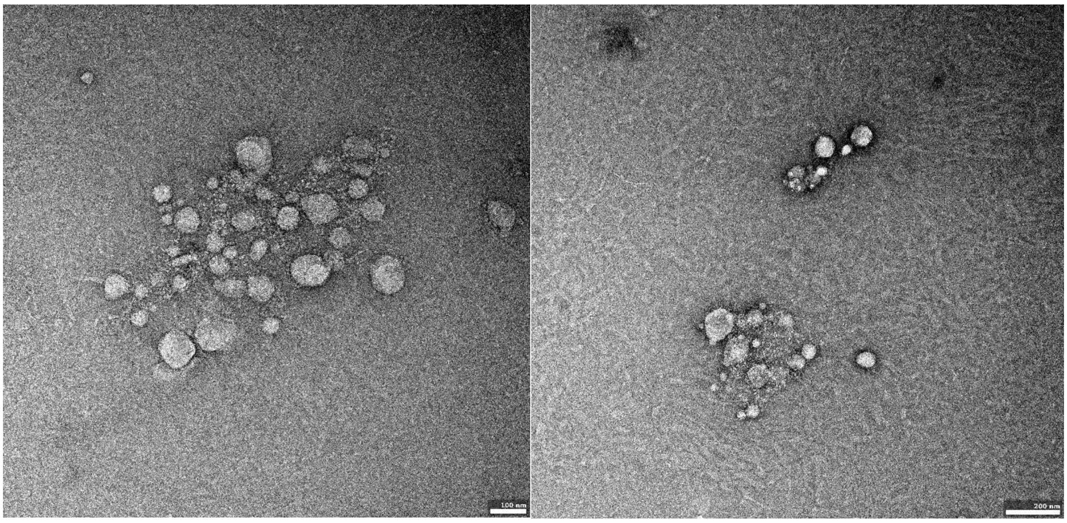

Supplementary Figure 3. TEM images of LpEVs shown at a wider field of view.

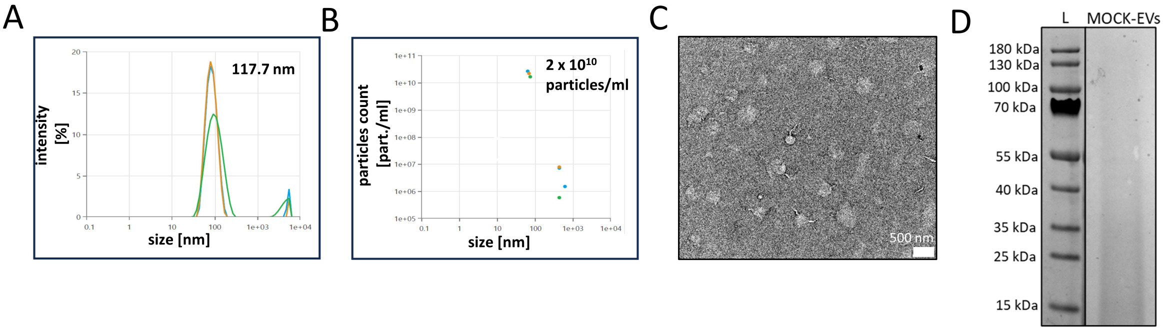

Supplementary Figure 4. Analysis of MOCK-EVs. (A) Particle size distribution of MOCK-EVs measured using a Zetasizer. (B) Particle concentration of MOCK-EVs measured using a Zetasizer. (C) TEM images of MOCK-EVs. (D) SDS–PAGE analysis of a 20 μL MOCK-EV sample.

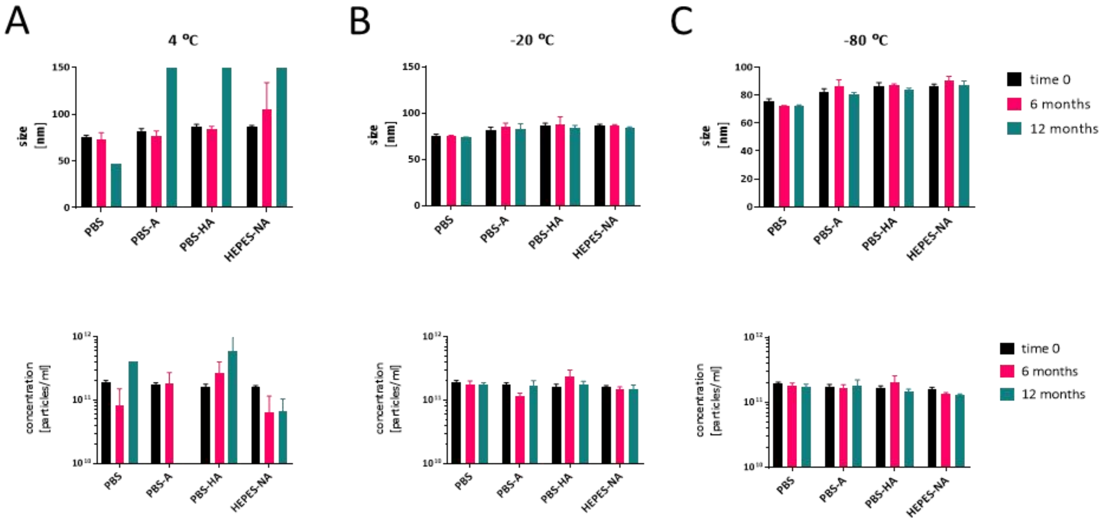

Supplementary Figure 5. Long-term stability of LpEVs under different buffer and temperature conditions. Particle size and concentration of LpEV samples stored at (A) 4°C, (B) -20°C, and (C) -80°C. Buffer composition: PBS, phosphate-buffered saline; PBS-A, PBS with 0.2% BSA; PBS-HA, PBS-A with HEPES; HEPES-NA, HEPES with 0.9% NaCl and 0.2% BSA.

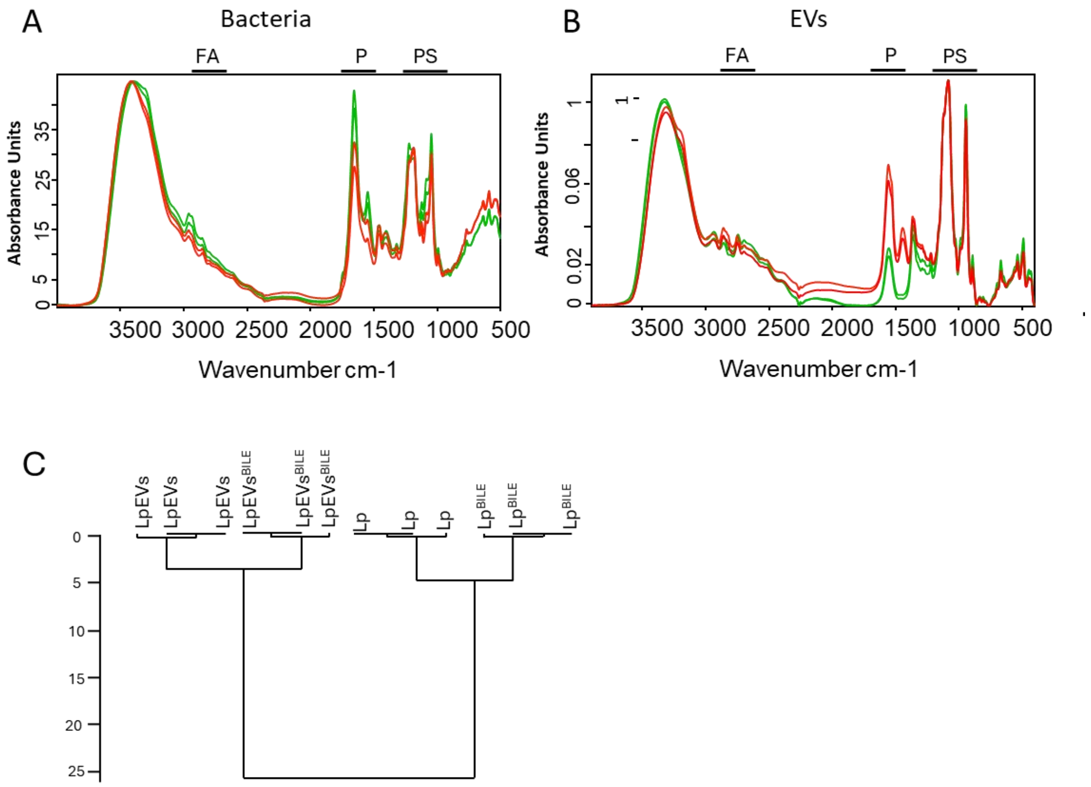

Supplementary Figure 6. Metabolic fingerprints of bacteria and EVs after cultivation in bile. Baseline- and vector-normalized spectra from two additional biological replicates of *L. plantarum* (green) and *L. plantarum*^{BILE} (red) (A) as well as LpEVs (green) and LpEVs^{BILE} (red) (B) are shown. (C) HCA of the entire spectral range. FA, fatty acids; P, proteins; PS, polysaccharides.

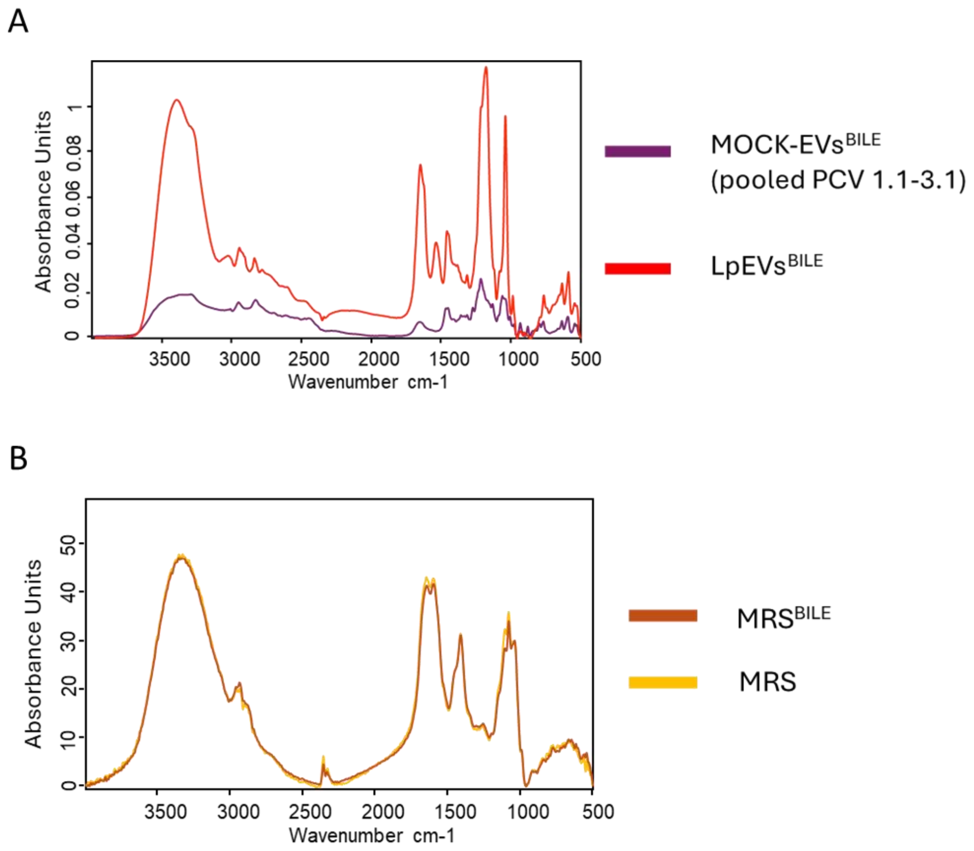

Supplementary Figure 7. FTIR spectra of control samples. (A) Representative FTIR spectra of pooled 1.1-1.3 SEC MOCK-EVs^{BILE} fractions. (B) Representative FTIR spectra of MRS- T with and without 0.244% bile.

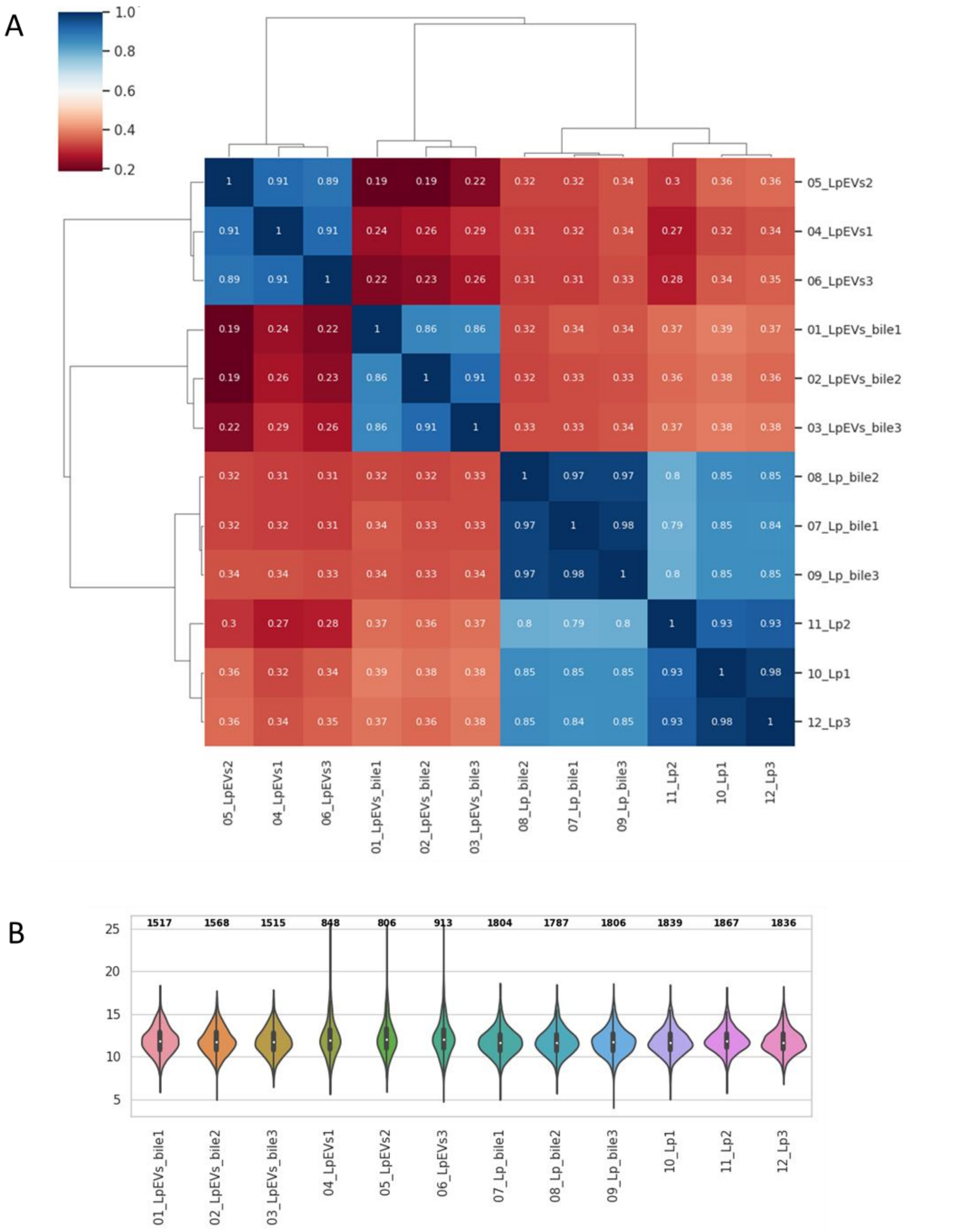

Supplementary Figure 8. Quality control data for proteomic analysis. (A) Cluster map of pairwise Pearson correlation coefficients between biological and technical replicates (distance metric: Euclidean; clustering method: average linkage). (B) Violin plots showing the number of observed values.

Supplementary Table 2. STRING analysis of protein–protein interaction networks

Biological Process (Gene Ontology)					
GO-term	description	count in network	strength	signal	false discovery rate
GO:0072524	Pyridine containing compound metabolic process	13 of 14	0.51	0.31	0.0459
GO:0006400	tRNA modification	18 of 23	0.43	0.32	0.0360
GO:0006364	rRNA processing	17 of 22	0.43	0.30	0.0449
GO:0009451	RNA modification	30 of 42	0.39	0.41	0.0097
GO:0034470	ncRNA processing	42 of 61	0.38	0.53	0.0013
GO:0008033	tRNA processing	24 of 35	0.37	0.33	0.0280
GO:0006396	RNA processing	43 of 65	0.36	0.50	0.0019
GO:0034660	ncRNA metabolic process	57 of 89	0.34	0.59	0.00029
GO:0006399	tRNA metabolic process	39 of 63	0.33	0.40	0.0097
GO:0006767	Water-soluble vitamin metabolic process	26 of 42	0.33	0.30	0.0410
GO:0016070	RNA metabolic process	71 of 128	0.28	0.52	0.00077
GO:1901615	Organic hydroxy compound metabolic process	35 of 63	0.28	0.30	0.0360
GO:0009165	Nucleotide biosynthetic process	47 of 88	0.27	0.34	0.0192
GO:0019438	Aromatic compound biosynthetic process	87 of 178	0.23	0.44	0.0023
GO:0034654	Nucleobase-containing compound biosynthetic process	67 of 137	0.23	0.35	0.0132

GO:0006753	Nucleoside phosphate metabolic process	62 of 127	0.23	0.33	0.0190
GO:1901362	Organic cyclic compound biosynthetic process	103 of 216	0.22	0.47	0.0012
GO:0018130	Heterocycle biosynthetic process	96 of 198	0.22	0.47	0.0013
GO:0055086	Nucleobase-containing small molecule metabolic process	71 of 149	0.22	0.35	0.0145
GO:0009117	Nucleotide metabolic process	58 of 121	0.22	0.31	0.0268
GO:0090407	Organophosphate biosynthetic process	58 of 125	0.20	0.28	0.0381
GO:0046483	Heterocycle metabolic process	242 of 544	0.19	0.68	1.88e-07
GO:0044281	Small molecule metabolic process	232 of 519	0.19	0.68	2.49e-07
GO:0019637	Organophosphate metabolic process	85 of 188	0.19	0.34	0.0145
GO:1901360	Organic cyclic compound metabolic process	250 of 568	0.18	0.67	1.88e-07
GO:0006725	Cellular aromatic compound metabolic process	237 of 534	0.18	0.67	2.49e-07
GO:0006139	Nucleobase-containing compound metabolic process	209 of 472	0.18	0.62	3.25e-06
GO:0090304	Nucleic acid metabolic process	134 of 316	0.17	0.39	0.0044
GO:0019752	Carboxylic acid metabolic process	102 of 240	0.17	0.32	0.0192

GO:0044283	Small molecule biosynthetic process	97 of 228	0.17	0.31	0.0217
GO:0044271	Cellular nitrogen compound biosynthetic process	125 of 301	0.16	0.34	0.0127
GO:0006796	Phosphate-containing compound metabolic process	121 of 291	0.16	0.33	0.0142
GO:0043436	Oxacid metabolic process	103 of 243	0.16	0.32	0.0192
GO:0006355	Regulation of transcription, DNA-templated	105 of 252	0.16	0.31	0.0227
GO:0034641	Cellular nitrogen compound metabolic process	257 of 627	0.15	0.57	1.24e-05
GO:0006793	Phosphorus metabolic process	125 of 306	0.15	0.32	0.0179
GO:0019219	Regulation of nucleobase-containing compound metabolic process	106 of 257	0.15	0.30	0.0257
GO:0050794	Regulation of cellular process	135 of 340	0.14	0.31	0.0217
GO:0006090	Regulation of primary metabolic process	111 of 277	0.14	0.28	0.0360
GO:0051171	Regulation of nitrogen compound metabolic process	111 of 276	0.14	0.28	0.0360
GO:0031326	Regulation of cellular biosynthetic process	109 of 270	0.14	0.28	0.0360
GO:0031323	Regulation of cellular metabolic process	111 of 276	0.14	0.28	0.0360

GO:0010556	Regulation of macromolecule biosynthetic process	109 of 270	0.14	0.28	0.0360
GO:0006082	Organic acid metabolic process	108 of 268	0.14	0.28	0.0360
GO:0071704	Organic substance metabolic process	526 of 1352	0.13	0.73	1.16e-12
GO:0044237	Cellular metabolic process	456 of 1179	0.13	0.67	2.82e-09
GO:0065007	Biological regulation	159 of 410	0.13	0.31	0.0192
GO:0019222	Regulation of metabolic process	113 of 286	0.13	0.27	0.0417
GO:0060255	Regulation of macromolecule metabolic process	112 of 284	0.13	0.26	0.0449
GO:0010468	Regulation of gene expression	110 of 279	0.13	0.26	0.0469
GO:0008152	Metabolic process	584 of 1512	0.12	0.76	5.17e-15
GO:0044238	Primary metabolic process	433 of 1128	0.12	0.64	4.16e-08
GO:0050789	Regulation of biological process	149 of 387	0.12	0.29	0.0267
GO:0006807	Nitrogen compound metabolic process	350 of 930	0.11	0.52	3.34e-05
GO:0009058	Biosynthetic process	233 of 627	0.11	0.34	0.0097
GO:0044249	Cellular biosynthetic process	217 of 579	0.11	0.34	0.0102
GO:1901576	Organic substance biosynthetic process	221 of 607	0.10	0.30	0.0217
GO:1901564	Organonitrogen	215 of	0.09	0.27	0.0360

	compound metabolic process	600			
Cellular component (Gene Ontology)					
GO-term	Type/description	count in network	strength	signal	false discovery rate
GO:0005829	Cytosol	147 of 299	0.23	0.68	1.65e-06
GO:0005737	Cytoplasm	519 of 1137	0.20	0.96	3.29e-27
GO:0005622	Intracellular anatomical structure	543 of 1232	0.18	0.93	2.86e-26
GO:0110165	Cellular anatomical entity	741 of 2390	0.03	0.32	0.0092
Local Network Cluster (STRING)					
CL:453	Mixed, incl. Catalytic activity, acting on a nucleic acid, and DNA repl...	58 of 103	0.29	0.31	0.0332
KEGG Pathways					
lpl01100	Metabolic pathways	209 of 510	0.15	0.48	0.00031
Subcellular Localization (COMPARTMENTS)					
GOCC:0043231	Intracellular membrane-bounded organelle	58 of 116	0.24	0.30	0.0320
GOCC:0005829	Cytosol	189 of 416	0.20	0.63	3.00e-06
GOCC:0005737	Cytoplasm	382 of 860	0.19	0.84	1.22e-14
GOCC:0005622	Intracellular	508 of 1235	0.15	0.81	1.63e-16
Annotated Keywords (UniProt)					
KW-0285	Flavoprotein	24 of 30	0.44	0.42	0.0098

KW-0819	tRNA processing	18 of 23	0.43	0.31	0.0435
KW-0436	Ligase	50 of 82	0.32	0.43	0.0052
KW-0560	Oxidoreductase	89 of 181	0.23	0.41	0.0052
KW-0804	Transcription	64 of 142	0.19	0.27	0.0458
KW-0963	Cytoplasm	115 of 262	0.18	0.39	0.0052
KW-0238	DNA-binding	82 of 185	0.18	0.31	0.0250
KW-0378	Hydrolase	146 of 350	0.16	0.38	0.0052
KW-0808	Transferase	177 of 478	0.11	0.26	0.0435
